# Immunogenicity of Fractional Doses of Tetravalent A/C/Y/W135 Meningococcal Polysaccharide Vaccine: Results from a Randomized Non-Inferiority Controlled Trial in Uganda

**DOI:** 10.1371/journal.pntd.0000342

**Published:** 2008-12-02

**Authors:** Philippe J. Guerin, Lisbeth M. Næss, Carole Fogg, Einar Rosenqvist, Loretxu Pinoges, Francis Bajunirwe, Carolyn Nabasumba, Ray Borrow, Leif O. Frøholm, Salah Ghabri, Vincent Batwala, Rogers Twesigye, Ingeborg S. Aaberge, John-Arne Røttingen, Patrice Piola, Dominique A. Caugant

**Affiliations:** 1 Epicentre, Paris, France; 2 Division of Infectious Disease Control, Norwegian Institute of Public Health, Oslo, Norway; 3 Centre for Prevention of Global Infections, University of Oslo, Oslo, Norway; 4 Mbarara University of Science and Technology, Mbarara, Uganda; 5 Health Protection Agency, Manchester, United Kingdom; 6 Norwegian Knowledge Centre for the Health Services, Oslo, Norway; 7 Department of Oral Biology, University of Oslo, Oslo, Norway; Sabin Vaccine Institute, United States of America

## Abstract

**Background:**

*Neisseria meningitidis* serogroup A is the main causative pathogen of meningitis epidemics in sub-Saharan Africa. In recent years, serogroup W135 has also been the cause of epidemics. Mass vaccination campaigns with polysaccharide vaccines are key elements in controlling these epidemics. Facing global vaccine shortage, we explored the use of fractional doses of a licensed A/C/Y/W135 polysaccharide meningococcal vaccine.

**Methods and Findings:**

We conducted a randomized, non-inferiority trial in 750 healthy volunteers 2–19 years old in Mbarara, Uganda, to compare the immune response of the full dose of the vaccine versus fractional doses (1/5 or 1/10). Safety and tolerability data were collected for all subjects during the 4 weeks following the injection. Pre- and post-vaccination sera were analyzed by measuring serum bactericidal activity (SBA) with baby rabbit complement. A responder was defined as a subject with a ≥4-fold increase in SBA against a target strain from each serogroup and SBA titer ≥128. For serogroup W135, 94% and 97% of the vaccinees in the 1/5- and 1/10-dose arms, respectively, were responders, versus 94% in the full-dose arm; for serogroup A, 92% and 88% were responders, respectively, versus 95%. Non-inferiority was demonstrated between the full dose and both fractional doses in SBA seroresponse against serogroups W135 and Y, in total population analysis. Non-inferiority was shown between the full and 1/5 doses for serogroup A in the population non-immune prior to vaccination. Non-inferiority was not shown for any of the fractionate doses for serogroup C. Safety and tolerability data were favourable, as observed in other studies.

**Conclusions:**

While the advent of conjugate A vaccine is anticipated to largely contribute to control serogroup A outbreaks in Africa, the scale-up of its production will not cover the entire “Meningitis Belt” target population for at least the next 3 to 5 years. In view of the current shortage of meningococcal vaccines for Africa, the use of 1/5 fractional doses should be considered as an alternative in mass vaccination campaigns.

**Trial Registration:**

ClinicalTrials.gov NCT00271479

## Author Summary

Meningitis are infections of the lining of the brain and spinal cord and can cause high fever, blood poisoning, and brain damage, as well as result in death in up to 10% of cases. Epidemics of meningitis occur almost every year in parts of sub-Saharan Africa, throughout a high-burden area spanning Senegal to Ethiopia dubbed the “Meningitis Belt.” Most epidemics in Africa are caused by *Neisseria meningitidis* (mostly serogroup A and W135). Mass vaccination campaigns attempt to control epidemics by administering meningococcal vaccines targeted against these serogroups, among others. However, global shortages of these vaccines are currently seen. We studied the use of fractional (1/5 and 1/10) doses of a licensed vaccine to assess its non-inferiority compared with the normal full dose. In a randomized trial in Uganda, we found that immune response and safety using a 1/5 dose were comparable to full dose for three serogroups (A, Y, W135), though not a fourth (C). In light of current shortages of meningococcal vaccines and their importance in fighting meningitis epidemics around the world, we suggest fractional doses be taken under consideration in mass vaccination campaigns.

## Introduction

Sub-Saharan African countries in the “Meningitis Belt,” situated between Ethiopia and Senegal, face epidemics of meningococcal meningitis almost every year [1]. Following the current World Health Organization (WHO) recommendation, mass vaccination campaigns with polysaccharide meningococcal vaccine are implemented solely to control the spread of the epidemic [2]. Until recently, *Neisseria meningitidis* serogroup A has been the main organism causing those epidemics, while other serogroups play a minor epidemiological role.

Following W135 outbreaks in Saudi Arabia in 2000 and 2001, cases of N. meningitidis serogroup W135 were reported in Burkina Faso in 2001, resulting in the first large W135 epidemic in that country in 2002 [3],[4]. This outbreak raised serious concerns regarding the availability of a vaccine protecting against that serogroup, i.e., a tetravalent A/C/Y/W135 polysaccharide vaccine (PSV). Mass vaccination of the population in Burkina Faso with the tetravalent PSV was not possible because of the global shortage in supply, in addition to its cost. In 2003, GlaxoSmithKline began producing a trivalent A/C/W135 polysaccharide vaccine for approximately USD1.50/dose, which was used in Burkina Faso in another epidemic the same year [5].

Since then however, availability and affordability of the tetravalent or trivalent polysaccharide vaccines remain uncertain every year. The production of the bivalent A/C polysaccharide vaccine has been considerably reduced since 2005 and the quantity of vaccines to be produced in the next 3 to 5 years is uncertain [6],[7]. In case of simultaneous large outbreaks in different countries, the supply of meningococcal PSV for the coming meningitis seasons is unlikely to be sufficient to cover vaccination needs (Perea W., WHO, personal communication, March 2008). Conjugate meningococcal vaccines, are not expected to be available and affordable in large quantities to cover the need for Africa over the next several years [7]–[9].

The current dose of the licensed tetravalent PSV developed in the 1970s contains 50 µg of each polysaccharide component. Studies in the 1970s and 1980s have shown that lower doses of polysaccharide were as effective as 50 µg in inducing bactericidal antibody levels that should be protective against disease in adults in the US [10]–[14].

To test if fractionate doses might also be protective in an African population and in younger age groups, we conducted a clinical vaccine trial in Uganda to evaluate the potential use of fractional doses of meningococcal tetravalent PSV to control disease outbreak caused by *N. meningitidis*. The study population selected for the trial was 2–19 years of age, i.e., the population at highest risk of the disease and the primary target of mass vaccination campaigns in Africa during epidemics [15].

## Methods

### Study design and population

The study design was a randomized, single-blind controlled trial. Three arms were defined in the trial: group 1 received a dose of 50 µg of each component of tetravalent PSV, i.e., a full dose of the licensed vaccine; group 2 received a 1/5 volume of tetravalent PSV (10 µg of each component); and group 3 received a 1/10 volume of tetravalent PSV (5 µg of each component).

The study was conducted in the rural area of Kinoni, Rwampara County, Mbarara District, Uganda. This location was chosen on the basis of the following criteria: i, this area had not experienced recent epidemics of meningococcal meningitis; ii, the study population was considered to be stable; iii, the health subdistrict was considered a suitable site for this interventional study because it has a long-standing collaboration with Mbarara University, Department of Community Health.

The recruitment of participants for the clinical trial was done on a voluntary basis. Volunteers aged 2–19 years old were recruited in proportions matching the Ugandan age distribution of the 2–19 years old extracted from the “2002/03 Uganda National Household Survey.” Volunteers were residents of Mbarara district, living within a 15-km radius of the vaccination site, with no plans of moving from the area during the study period. Community awareness meetings were held with local leaders and field workers from the study team, who then went house to house to get a list of people who were willing to participate. Participants came to the study site on a planned date. Refusal rates were not recorded in order to avoid unnecessary pressure on the communities.

### Objectives and outcomes

This study aimed to demonstrate non-inferiority in the immune response of doses corresponding to 1/5 and/or 1/10 of the amount of the full dose of a licensed A/C/Y/W135 polysaccharide vaccine (Menomune, Sanofi Aventis) and to evaluate the tolerability of these vaccinations. The primary endpoint was the proportion of responders defined by immunogenicity criteria at four weeks after vaccination based on SBA titers. The secondary endpoint considered the IgG response (Elisa).

### Sample size calculation

The sample size was calculated by choosing a one-sided 0.05 level of significance and power of 80%. Expecting equal proportions of responders in all groups given the vaccine being 80%, and assuming a non-inferiority margin of 10%, this gave a required sample size n of 198 persons in each group. Because the reference group (full dose) was used for two comparisons, a correction of (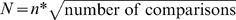) was applied [16], bringing that group to 280. The calculations have been performed using nQuery Advisor.

### Randomization and allocations

Following consent and a clinical examination, each subject was randomly allocated to one of the 3 dosage groups. The allocation schedule was computer-generated, using a block randomization method, stratified by age group (2 to 4; 5 to 9; 10 to 14 and 15 to 19 years). The researchers responsible for seeing the volunteers allocated the next available number on entry into the trial. The vaccination was given subcutaneously using low-volume syringes (0.5mL BD Micro-Fine insulin syringes), by the same nurse throughout the study, without participant knowledge of the dosage received. A single dose vaccine Menomune vial was used per volunteer, numbered with the study number and stored after vaccination. A full dose injection corresponded to 0.5ml of the vaccine, 1/5 of the dose corresponded to 0.1ml and 1/10 of the dose to 0.05ml.

### Safety

Volunteers were observed for 1 hour following vaccination for adverse events. Safety and tolerability data were collected for all volunteers during the 4 weeks following the injection. Safety data were collected during weekly interviews. The intensity of the adverse events was evaluated by clinicians, members of the study team and classified as “mild,” “moderate,” or “severe” using the Common Toxicity Criteria (CTC) grading (http://ctep.cancer.gov/reporting/CTC-3.html, US National Cancer Institute).

### Laboratory analysis

Serum samples (10 mL of whole blood) were collected from each volunteer immediately before vaccination and 4 weeks later, stored at −80°C from the trial to the laboratories. Assays were carried out blinded at the Norwegian Institute of Public Health (NIPH). Immune responses to the different doses of the TPSV were analyzed in serum bactericidal assays (SBA) and enzyme-linked immunosorbent assays (ELISA). SBA was performed against four target strains of the A, C, W135, and Y serogroups: A: F8238 (4/21:P1.20,9); C: C11 (16:P1.7-1,1); W135: M01240070 (NT:P1.18-1,3); and Y: M00242975 (2a:P1.5,2). Heat-inactivated test sera were diluted 2-fold in microtiter plates (starting at serum dilution of 1:4) and incubated for 60 min with bacteria and baby rabbit complement (Pel-Freeze) before plating onto agar plates [17]. Colony-forming units were counted (Sorcerer colony counter, Perceptive Instruments), and bactericidal antibody titers were expressed as the reciprocal of the final serum dilution giving ≥50% killing compared with controls (inactive complement/no test serum). External quality control of SBA measurements was performed by Manchester Health Protection Agency (HPA) by analyzing in parallel approximately 10% of samples taken before vaccination and four weeks later. IgG antibodies to each separate polysaccharide A, C, Y, and W135 were measured in ELISA as described by Carlone *et al.* [18] and modified according to Joseph *et al.* using the CDC 1992 standard (NIBSC code 99/706) [19].

### Carriage study

Tonsillo-pharyngeal samples were collected from the volunteers before vaccination and four weeks later. The technique and results of this carriage study are published elsewhere [20]. Volunteers found to be carriers of *N. meningitidis* of a homologous serogroup at any time between the vaccination and four weeks later were excluded from the analysis of response to that polysaccharide.

### Statistical analysis

For computational purposes, titers <4 were assigned a value of 2. A subject with SBA titer ≥128 was defined as putatively protected [21]. The Modified Intention To Treat (MITT) population included all randomized and exposed subjects with a defined SBA titer before vaccination and four weeks later. The Per Protocol (PP) population excluded subjects from the MITT presenting protocol violation. Some immunogenicity measures were not planned and described in the statistical analysis of the protocol. For the benefit of the study, the scientific committee coordinating the trial suggested additional statistical analyses: i, the principal criteria to define a responder was reinforced, as not only a 4-fold or greater increase in antibody titer between pre- and post-immunization sera, but also an SBA titer ≥128 four weeks after vaccination; ii, we also considered an exploratory population of the MITT, namely the “non-immune population” before vaccination, defined as individuals with SBA titers <128 before vaccination, which is considered the threshold of non-immunity [21]–[25]. Baseline characteristics were summarized by treatment groups using descriptive statistics (Geometric Mean Titer [GMT] and Geometric Mean Concentration [GMC)] were used for the analysis of the SBA titers and IgG concentrations). McNemar's test was used to compare matched pair titer data before vaccination and four weeks later.

The proportion of adequate responses in each group was expressed as a percentage (“response to vaccine rate”). A 95% confidence interval was calculated for the observed difference in response proportion (full versus fractional dose), and if the upper limit was <10%, the fractional dose was considered non-inferior to the full dose. These analyses were performed on MITT, PP, and non-immune subsets of the MITT.

We performed a logistic regression to look at the impact of age among responders by serogroup and by arm. Age was considered in two groups of interest (≤5 and >5 years of age) knowing that in previous studies, eliciting an immune response under 5 was the most critical [11],[26].

Data were double-entered using Epidata 3.0 (The EpiData Association, Odense, Denmark). Statistical analyses were performed using STATA 9 (College Station, Texas, USA).

### Ethics

Written informed consent in the local language was obtained from the parents or guardians of every volunteer <18 years of age or by the volunteers themselves if >18 years. The study was approved by the Faculty Research and Ethics Committee of the Mbarara University of Science and Technology (MUST), the MUST Institutional Review Board, the Uganda National Committee of Science and Technology, and the Regional Committee for Medical Research Ethics in Norway. The trial was registered at Clinicaltrials.gov (NCT00271479).

## Results

### Study groups

**Figure 1:**
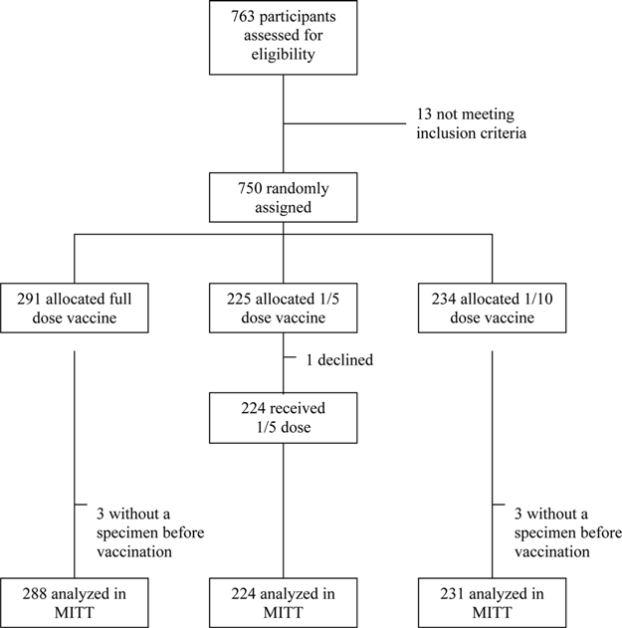
Consort flowchart.

Between 5 July 2004 and 22 September 2004, 763 volunteers from the Kinoni community in Mbarara, Uganda were screened (Figure 1). Among them, 750 volunteers were included, with 291 randomized to the full-dose vaccine arm, 225 to the 1/5-dose arm, and 234 to the 1/10-dose arm.

The demographic and serological baseline characteristics of the population at inclusion before vaccination are displayed in Table 1. For each serogroup, volunteers were not considered in the analyses if an SBA value was missing for either before vaccination or four weeks later. No differences were observed between arms for demographic and serological data.

**Table 1:** Demographic and serological baseline characteristics

		Full dose (n = 291)	1/5 dose (n = 224)*	1/10 dose (n = 235)*
**Age (years)**	Median (IQR)	9.1 (5.1–13.1)	9.1 (5.1–13.1)	9.1 (5.1–14.1)
**Sex**	Ratio (M/F)	0.89 (137/154)	1.06 (115/109)	0.90 (111/124)
**Weight (kg)**	Median (IQR)	25 (16–37)	25 (17–37)	25 (17–39)
**Height (cm)**	Median (IQR)	128.0 (107–146.5)	127.1 (110–146.5)	129.8 (108.5–149.4)
**SBA A titers**	GMT (GSD)	41.6 (21.6)	58.7 (20.9)	48.8 (21.0)
**GMC IgG** µ**g/mL ELISA A**	GMC (GSD)	2.6 (2.4)	2.8 (2.3)	2.4 (2.4)
**SBA W135 titers**	GMT (GSD)	7.1 (10.0)	6.0 (8.6)	7.1 (10.1)
**GMC IgG** µ**g/mL ELISA W135**	GMC (GSD)	2.7 (2.5)	2.9 (2.4)	2.9 (2.3)
**SBA C titers**	GMT (GSD)	2.7 (3.5)	2.8 (3.9)	3.5 (5.3)
**GMC IgG** µ**g/mL ELISA C**	GMC (GSD)	0.9 (2.4)	1.0 (2.4)	1.0 (2.3)
**SBA Y titers**	GMT (GSD)	2.2 (1.9)	2.7 (3.8)	2.2 (2.0)
**GMC IgG µg/mL ELISA Y**	GMC (GSD)	2.9 (2.4)	3.1 (2.4)	3.1 (2.3)

*: One patient randomized in the 1/5-dose group received 1/10 of the dose.

GSD: Geometric Standard Deviation.

Natural immunity toward *N. meningitidis* serogroups A, C, Y, and W135 before vaccination in the study population was measured by the proportion of volunteers with SBA titers ≥128 before vaccination: 51.4% (382/743) for serogroup A; 22.6% (168/744) for serogroup W135; 6.2% (45/729) for serogroup C, and 2.3% (17/741) for serogroup Y.

### Immunologic response

Protocol deviations leading to exclusion of population are described in Table 2. The primary end point, i.e. proportions of responders per arm and per analyses are reported in Table 3. For serogroup W135, 94.4% (168/178) of the non-immune, vaccinated subjects in the 1/5-dose arm, and 97.2% (172/177) in the 1/10-dose arm, were responders, compared with 93.7% (207/221) in the full-dose arm. For serogroup A, 92.2% (94/102) and 88.3% (98/111) of non-immune vaccinees in the 1/5- and 1/10-dose arms, respectively, were responders, compared with 94.6% (140/148) in the full-dose arm.

**Table 2:** Description of individual exclusions by population (MITT, PP, Safety population) and randomized group

Reason for exclusion	Number of volunteers	Randomized group	Excluded for
Received 1/10 instead of 1/5	1	1/5 dose	PP all serogroups
Withdrew consent before vaccination	1	1/10 dose	MITT, PP, Safety Analysis all serogroups
Mis-stratified*	3	2 in 1/5 dose and 1 in 1/10	PP all serogroups
Carrier of W135**	2	1in Full dose and 1 in 1/10 dose	PP for W135
Malnourished (weight/height <−2 Z-score)	1	Full dose	PP all serogroups

*: Allocated to the wrong age group.

**: Volunteers presenting a carriage of W135 between the vaccination and four weeks later.

**Table 3:** Proportion of responders per serogroup and per population and SBA GMT at 4 weeks after vaccination

Analyses	Dose Group	Proportion of Responders			SBA Titers four weeks after vaccination	
		n/N	%	95% CI	GMT	95% CI
Serogroup A						
MITT	Full	249/289	86.2	82.2–90.2	3607.1	2952.8–4406.3
	1/5 dose	173/224	77.2	71.7–82.7	2035.4	1600.1–2589.0
	1/10 dose	159/230	69.1	63.2 -75.0	1367.6	1083.1–1726.8
PP	Full	247/287	86.1	82.1–90.1	3612.6	2953.3–4419.1
	1/5 dose	172/222	77.5	72.0–83.0	2054.4	1612.2–2618.0
	1/10 dose	159/229	69.4	63.5–75.3	1369.3	1083.3–1730.7
Non immune	Full	140/148	94.6	91.0–98.2	1918.0	1426.0–2579.8
	1/5 dose	94/102	92.2	87.0–97.4	852.3	573.2–1267.5
	1/10 dose	98/111	88.3	82.4–94.2	754.1	495.3–1148.1
Serogroup W135						
MITT	Full	269/289	93.1	90.2–96.0	2190.3	1728.9–2774.6
	1/5 dose	212/224	94.6	91.7–97.5	2029.1	1573.7–2616.2
	1/10 dose	220/231	95.2	92.5–97.9	2422.7	1979.9–2964.6
PP	Full	267/286	93.4	90.5–96.3	2175.9	1714.2–2762.0
	1/5 dose	210/222	94.6	91.6–97.6	2041.6	1582.2–2634.5
	1/10 dose	219/229	95.6	93.0–98.2	2426.3	1979.7–2973.7
Non immune	Full	207/221	93.7	90.5–96.9	1539.5	1160.0–2043.2
	1/5 dose	168/178	94.4	91.0–97.8	1517.4	1129.3–2039.0
	1/10 dose	172/177	97.2	94.8–99.6	2008.3	1583.0–2547.9
Serogroup C						
MITT	Full	259/284	91.2	87.9–94.5	1168.3	911.0–1498.2
	1/5 dose	179/222	80.6	75.4–85.8	472.1	332.3–670.6
	1/10 dose	171/223	76.7	71.2–82.2	399.3	277.1–575.4
PP	Full	257/282	91.1	87.8–94.4	1175.1	914.9–1509.3
	1/5 dose	177/220	80.4	75.2–85.6	467.3	328.0–665.8
	1/10 dose	170/222	76.6	71.1–82.1	396.3	274.6–572.0
Non immune	Full	252/271	93.0	90.0–96.0	1108.5	858.4–1431.5
	1/5 dose	172/211	81.5	76.3–86.7	412.2	288.2–589.6
	1/10 dose	156/202	77.2	71.4–83.0	315.6	214.8–463.8
Serogroup Y						
MITT	Full	242/286	84.6	80.4–88.8	936.2	673.7–1301.0
	1/5 dose	185/224	82.6	77.7–87.5	772.7	529.2–1128.2
	1/10 dose	194/231	84.0	79.3–88.7	822.6	569.8–1187.5
PP	Full	240/284	84.5	80.3–88.7	924.2	663.8–1286.7
	1/5 dose	183/222	82.4	77.4–87.4	768.3	524.4–1125.6
	1/10 dose	193/230	83.9	79.2–88.6	816.8	565.1–1180.8
Non immune	Full	238/282	84.4	80.2–88.6	916.8	657.3–1278.8
	1/5 dose	175/214	81.8	76.7–86.9	687.5	466.1–1014.1
	1/10 dose	191/228	83.8	79.0–88.6	798.1	551.1–1155.7

Non-inferiority was demonstrated for serogroups W135 and Y (full dose versus each fractional dose in MITT analyses), but was statistically rejected for serogroups A and C (Table 4). When analyzing only the non-immune population, non-inferiority was also demonstrated for full versus 1/5 doses for serogroups A (2.4% [95% confidence interval, −3.9 to 8.8%]), W135 (−0.7% [95% confidence interval, −5.4 to 3.9%]), and Y (2.6% [95% confidence interval, −4.1 to 9.3%]), but not for serogroup C (11.5% [95% confidence interval, 5.4 to 17.5%]) (Table 4). When considering the response by age group (logistic regression), children under 5 had a lower chance of positive response compared to older ones for serogroup W135 (significant only for full dose arm), serogroup C (significant for full dose and 1/5 dose arms) and for serogroup Y (significant for 1/10 dose arm) (Table 5). For serogroup A, although not significant, fractional doses seem to elicit a better response in children under 5.

**Table 4:** Non-inferiority analysis results of SBA responders per serogroup and analyses

	Total population		Non-immune population	
**Serogroup A**	**MITT** (n = 743)		(n = 361)	
	Diff.	95%CI	Diff.	95%CI
**Full dose vs 1/5**	+8.9%	[+2.1%, +15.7%]	+2.4%	[−3.9%, +8.8%]
**Full dose vs 1/10**	+17.0%	[+9.8%, +24.2%]	+6.3%	[−0.7%, +13.3%]
**Serogroup W135**	**MITT** (n = 744)		(n = 576)	
	Diff.	95%CI	Diff.	95%CI
**Full dose vs 1/5**	−1.6%	[−5.7%, +2.6%]	−0.7%	[−5.4%, +3.9%]
**Full dose vs 1/10**	−2.2%	[−6.2%, +1.9%]	−3.5%	[−7.5%, +0.5%]
**Serogroup C**	**MITT** (n = 729)		(n = 684)	
	Diff.	95%CI	Diff.	95%CI
**Full dose vs 1/5**	+10.6%	[+4.4%;+16.7%]	+11.5%	[+5.4%;+17.5%]
**Full dose vs 1/10**	+14.5%	[+8.1%;+21.0%]	+15.8%	[+9.2% ; +22.3%]
**Serogroup Y**	**MITT** (n = 741)		(n = 724)	
	Diff.	95%CI	Diff.	95%CI
**Full dose vs 1/5**	+2.0%	[−4.5%;+8.5%]	+2.6%	[−4.1%;+9.3%]
**Full dose vs 1/10**	+0.6%	[−5.7%;+6.9%]	+0.6%	[−5.8%;+7.0%]

**Table 5:** Logistic regression results of age effect on responder per serogroup and per arm–MITT population

Serogroups	Full dose		1/5 dose		1/10 dose	
Age>5 vs. ≤5 years old	OR	(95% CI)	OR	(95% CI)	OR	(95% CI)
**Serogroup A**	2.07	(0.99;4.31)	0.62	(0.27; 1.42)	0.79	(0.39; 1.61)
**Serogroup W135**	2.78	(1.08; 7.15)	2.81	(0.85; 9.28)	0.37	(0.05; 2.95)
**Serogroup C**	3.37	(1.44; 7.87)	2.83	(1.36; 5.87)	1.66	(0.81; 3.44)
**Serogroup Y**	1.99	(0.98; 4.05)	1.84	(0.85; 3.98)	2.46	(1.14; 5.30)

**Figure 2:**
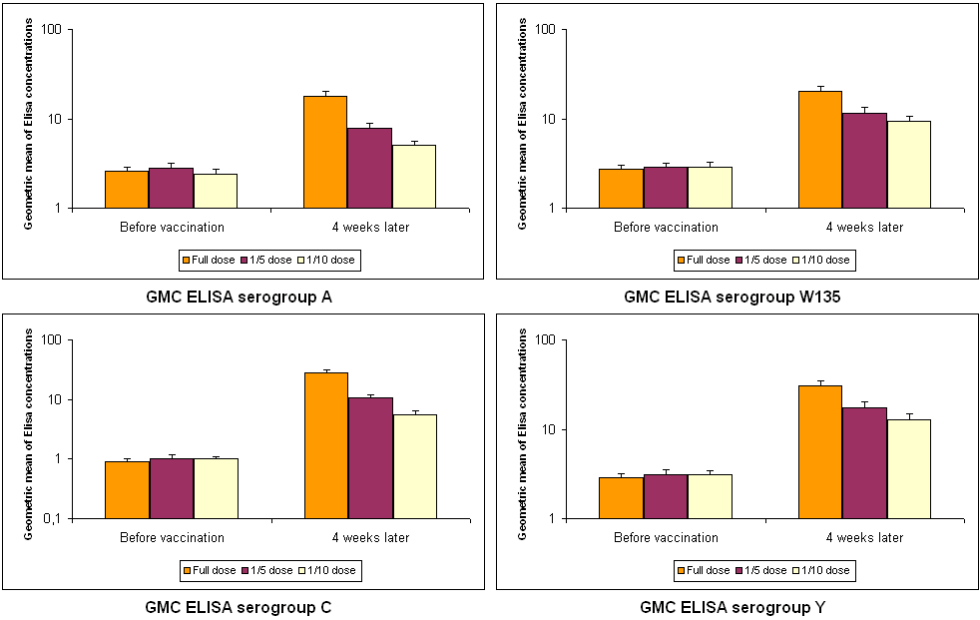
ELISA IgG concentrations per serogroup and per arm before the vaccination and four weeks later in the MITT population (GMC with superior limit of 95%CI). GMC = geometric mean concentration. *P*<0.001 for all comparisons between the vaccination and four weeks later for all serogroups.

The secondary immunogenicity criterion based on ELISA data is reported on Figure 2. For each serogroup and each dose of vaccine, the geometric means of IgG concentrations showed no difference between arms before vaccination but a significant difference four weeks later with full dose greater than both 1/5 and 1/10 doses. Statistically significant differences were observed between the vaccination and four weeks later for each dose and each serogroup (p<0.0001 for all comparisons).

### Adverse events

A total of 158 volunteers reported at least one adverse event during the 4 weeks after vaccination (171 total adverse events, Table 6). No significant statistical difference was observed among the three dose arms (χ^2^ test, p = 0.42). The most commonly reported adverse events were upper respiratory tract infections (URTI) (57%) and malaria (20%). Five severe adverse events were recorded: one severe case of malaria, one severe episode of seizures, and 3 severe URTI, but these events were not considered to be related to the vaccination. Three adverse events considered “probably related” were reported and classified as mild (2 subjects with fever and 1 with headache).

**Table 6:** Distribution of adverse events following vaccination

Distribution of adverse events per week post-vaccination	Attendance at schedule visits n/N (%)	Full dose	1/5 dose	1/10 dose	Total
First week	745/749 (99.5%)	25	16	18	59 (34.5%)
Second week	746/749 (99.6%)	22	11	18	51 (29.8%)
Third week	746/749 (99.6%)	12	5	6	23 (13.5%)
Fourth week	749/749 (100%)	17	11	10	38 (22.2%)
**Total**		76 (44.4%)	43 (25.1%)	52 (30.4%)	171 (100%)

### Quality control

External quality control of the SBA titer measurements showed no significant difference with regard to responders for serogroup A (McNemar pair matched test, p =  0.63), serogroup C (p = 0.06), and serogroup Y (p = 0.41). For serogroup W135, the difference was statistically significant (p<0.001).

## Discussion

SBA is the accepted correlate of protection for meningococcal disease. In the MITT analysis of this study, non-inferiority was demonstrated between full and 1/5 and 1/10 fractional doses of TPSV in SBA response against the meningococcal serogroups W135 and Y. Non-inferiority was only shown between the full and 1/5 doses for serogroup A in the pre-vaccination, non-immune population. Non-inferiority was rejected for serogroup C in all analyses. Safety and tolerability data were favourable, as observed with TPSV in other studies [27],[28].

In analyzing the proportion of responders per serogroup, we observed a decline in response for serogroup A and C from the full versus 1/5 dose, and this decrease was accentuated versus the 1/10 dose. For serogroup A, which is the most important serogroup to protect against in sub-Saharan Africa, the response in the MITT analysis decreased from 86% to 77%. Several elements must be considered in the interpretation of these results. A notable proportion of volunteers (51.4%) had high SBA titers against serogroup A prior to vaccination, presumably resulting from natural immunity. In demonstrating non-inferiority between the full and 1/5 dose groups in the non-immune population, the difference in responses occurred mainly in the naturally immune subgroup. These results suggest that the full dose may elicit higher increase in SBA titers for subjects with pre-vaccination SBA titers ≥128 compared with 1/5 of the dose. However, assuming that a post-vaccination SBA titer ≥128 is a proxy for vaccine efficacy, we believe that 1/5 of the dose induced an acceptable increase of SBA for non-immune populations, although it did not strictly meet the criteria we designed for the total population. When considering the response for children under five, overall fractional doses do not affect the chance of response compared to full dose. For serogroup A, the response could be possibly better in children under five with fractional doses, though the study was not powered to demonstrate this hypothesis.

For all serogroups, the IgG concentrations decreased with fractional doses. However, the SBA titer/IgG ratios showed similar results between arms for all serogroups (data not shown), indicating a higher proportion of bactericidal antibodies in fractional doses. This could be due to differences in antibody avidity, though this hypothesis would require further studies. In an epidemic response setting, the goal of a mass vaccination campaign is short term immunity-basically protection through to the end of the epidemic season. Therefore, longer duration of protection (presumably predicted by higher titers) is a less important issue.

Licensed meningococcal polysaccharide vaccines are known to confer an immunity of short duration (2–3 years) and are therefore not recommended in expanded vaccination programs [6],[29]. But this characteristic may not impact the use of fractional dosing in a reactive mass vaccination campaign aimed at preventing further new cases during an ongoing epidemic. Study subjects in this trial were followed up to 2 years, and the duration of protection will be addressed later on.

Several potential limitations of this study must be addressed. Tolerability data were excellent; however, the weekly visits between the vaccination and four weeks later may not have been optimal to capture adverse events often occurring in the first days after vaccination. HIV testing was not systematically performed. Considering the epidemiological indicators of HIV in the adult population aged 15–49 years (HIV prevalence rate 6.7% [5.7–7.6]) [30], and the exclusion criteria of known or suspected cases in our study population, the impact of HIV is unlikely to be noticeable. Injections of fractional doses with “insulin syringes” were considered relatively simple to perform in the field for the 1/5 (0.1 mL) dose, but the 1/10 (0.05 mL) dose was more difficult to inject. Such difficulty may have hampered the delivery of the 1/10 fractional dose. This evaluation was based on the informal evaluation from the study team. Considering the absence of difficulties to inject 1/5 of the dose providing the use of appropriate syringes and training, health workers engaged in an outbreak response during an epidemic should not faced major problems to implement this vaccination. The unexpected high background rate of immunity to serogroup A in the study population has been a constraint to demonstrate the impact of the vaccination for this serogroup. Despite the fact that no large outbreak of meningococcal meningitis due to serogroup A had been declared in southern Uganda in the years prior to the study, it is likely that the strain was circulating in the region, following the outbreaks of serogroup A in neighbouring countries, Burundi and Rwanda in 2002 [31].

Quality control of the SBA titers showed satisfactory results for serogroups A, C, and Y. However, a discrepancy was found for the W135 serogroup. This discrepancy was found to be due to the use of a different strain between the two laboratories. Once repeated with same strain, there was no significant difference between the results of the two laboratories (p = 0.31). As the proportion of responders for serogroup W135 was the same in the two laboratories and the source of the discrepancy was identified, we believe that our overall results of serogroup W135 are validated.

Baby rabbit complement was used in the SBA assays in accordance with international standard protocols to evaluate polysaccharide vaccines against meningococcal disease, but SBA with human complement might be more relevant to elucidate the immune response after disease and vaccination. Additional insight would be gained by assaying these sera in a human complement SBA assay, and such analyses are ongoing.

The two prevailing serogroups that cause *N. meningitidis* epidemics in the African Meningitis Belt are A and W135, and serogroups C and Y are not presently reported as the causal agent of meningitis epidemics in the region [6]. The WHO states that problems regarding the availability and affordability of protective meningococcal vaccines over the coming years need to be addressed urgently [6]. A risk-benefit analysis of the use of fractional doses should guide decision-makers. Similar strategies with other vaccines have already proved successful [32]. Assuming 90%, short-term protection by the licensed meningococcal polysaccharide vaccines, and a conservative protection of 80% using a reduced 1/5 dose, the same amount of resources invested in vaccine purchase would protect 4.4 times more subjects. Although the cost of immunization is not a primary interest of this strategy in the context of a global shortage, the use of a fractional dose would decrease the cost per person vaccinated by approximately half (data not shown). While the advent of conjugate A vaccine will largely contribute to control serogroup A outbreaks in Africa, the scale-up of its production will not cover the entire “meningitis belt” target population over the next 3 to 5 years (Laforce M., Meningitis Vaccine Project, personal communication January 2008). Considering the current shortage of meningococcal vaccines for Africa and the prevalence of serogroups A and W135, the use of 1/5 fractional doses should be explored as an alternative strategy in mass vaccination campaigns.

## Supporting Information

Alternative Language Abstract S1 Translation of the Abstract into French by Philippe J. Guerin
(0.03 MB DOC)

Protocol S1 Final approved protocol
(1.03 MB PDF)

Checklist S1 Consort Checklist
(0.06 MB DOC)
