# Supplementary material for: Immunogenicity of Fractional Doses of Tetravalent A/C/Y/W135 Meningococcal Polysaccharide Vaccine: Results from a Randomized Non-Inferiority Controlled Trial in Uganda
Source: PLoS Negl Trop Dis. 2008 Dec 2;2(12):e342. doi: 10.1371/journal.pntd.0000342 (PMC2584372; doi:10.1371/journal.pntd.0000342)
Supplement: Alternative Language Abstract S1 — Translation of the Abstract into French by Philippe J. Guerin (0.03 MB DOC) [file pntd.0000342.s001.doc]

**Translation of the abstract into French by Guerin PJ**

Immunogénicité de doses fractionnées d’un vaccin méningococcique polysaccharidique tétravalent A/C/W135/Y : résultats d’une étude contrôlée de non-infériorité en Ouganda

Résumé
Introduction: Neisseria meningitidis sérogroupe A est le principal agent pathogène causant des épidémies de méningite en Afrique sub-saharienne. Au cours des dernières années, le sérogroupe W135 a également été à l'origine d’épidémies. Des campagnes de vaccination de masse avec les vaccins polysaccharidiques font parties de stratégies clefs dans la lutte contre ces épidémies. Face à la pénurie internationale de vaccins, nous avons exploré l'utilisation de doses fractionnées d'un vaccin commercialisé méningococcique polysaccharidique A/C/Y/W135.

Méthodes et résultats: Nous avons conduit une étude de non-infériorité randomisée sur 750 volontaires sains entre 2-19 ans dans la région de Mbarara, en Ouganda, afin de comparer la réponse immunitaire entre la pleine dose du vaccin versus des doses fractionnées (1/5 ou 1/10 de la pleine dose). Des données sur la tolérance et effets indésirables ont été recueillies chez l’ensemble des volontaires au cours des 4 semaines suivant l'injection. Des échantillons sanguins pré-et post-vaccination ont été analysés afin de mesurer l'activité bactéricide du sérum (ABS). Un répondeur à la vaccination a été défini comme un sujet présentant une augmentation ≥ 4 du titre d’ABS contre une souche cible pour chaque sérogroupe et un titre d’ABS≥ 128.
Pour le sérogroupe W135, 94% et 97% des sujets vaccinés ayant reçus respectivement 1/5 et 1/10 de la dose étaient répondeurs, contre 94% pour ceux ayant reçus la pleine dose ; Pour le sérogroupe A, 92% et 88% étaient répondeurs, contre 95%. La non-infériorité a été démontrée entre la pleine dose et les deux doses fractionnées pour les sérogroupes Y et W135, pour l’analyse en population totale. La non-infériorité a été établie entre la pleine dose et 1/5 de la dose dans la population non-immune avant la vaccination pour le sérogroupe A. La non-infériorité n'a pas été montrée pour les doses fractionnées pour le sérogroupe C. Les données de tolérance ont été très bonnes, comme observées dans d'autres études.

Conclusions: Bien que la commercialisation d'un vaccin conjugué A devrait largement contribuer à contrôler les épidémies de méningite à méningocoque à sérogroupe A en Afrique, les vaccines disponibles ne couvriront pas l'ensemble de la "ceinture méningitique" population cible pour au moins les 3 à 5 prochaines années. Compte tenu de la pénurie actuelle de vaccins méningococciques pour l'Afrique, l'utilisation de dose fractionnée à 1/5 devrait être considérée comme une alternative dans les campagnes de vaccination de masse.
(N° ClinicalTrials.gov, NCT00271479)
