# Supplementary material for: Immunogenicity of Fractional Doses of Tetravalent A/C/Y/W135 Meningococcal Polysaccharide Vaccine: Results from a Randomized Non-Inferiority Controlled Trial in Uganda
Source: PLoS Negl Trop Dis. 2008 Dec 2;2(12):e342. doi: 10.1371/journal.pntd.0000342 (PMC2584372; doi:10.1371/journal.pntd.0000342)
Supplement: Protocol S1 — Final approved protocol (1.03 MB PDF) [file pntd.0000342.s002.pdf]

Assay of the immunogenicity of fractional dose tetravalent A, C, Y, W135 meningococcal polysaccharide vaccine in Africa

Project proposal

2004 - 2007

Product: Tetravalent A, C, Y, W135 meningococcal polysaccharide vaccine (Menomune®)

Version: Mbarara University Committee Review –

Design: Non inferiority trial

Study Site: Mbarara University Teaching Hospital
Mbarara, Uganda

Sponsor: Norwegian Institute of Public Health (NIPH)
Globinf - University of Oslo, Norway
World Health Organisation
Médecins Sans Frontières (MSF)

Principal Investigator:
Philippe J Guerin
Epicentre
8 rue Saint Sabin
75011 Paris
Tel. +33.(0).1.40.21.28.48
E-mail: philippe.guerin@epicentre.msf.org

Co-Principal Investigators :
Laurence Ahoua
Epicentre
Uganda

Dominique A. Caugant
NIPH
Norway

Co-Investigators:
Ingeborg Aaberge (NIPH)
Carole Fogg (Epicentre Uganda)
Lisbeth Meyer Næss (NIPH)

Co-Investigator from Mbarara University:
Francis Bajunirwe
Department of Community Health
Mbarara University of Science and Technology (MUST)

Data Management: Epicentre

Pharmaceutical Support*:* Aventis Pasteur will provide tetravalent A, C, Y, W135 meningococcal polysaccharide vaccines

Protocol Development Team*:*
Philippe J Guerin (Epicentre)
Dominique Caugant (NIPH)
Ingeborg Aaberge (NIPH)

Einar Rosenqvist (NIPH)
E. A. Høiby (NIPH)
John-Arne Røttingen (NIPH)
Oddvar Frøholm (NIPH)
Laurence Ahoua (Epicentre)
Patrice Piola (Epicentre)
Eugene Ruzagira (Epicentre)
Joseph Babigumira (Epicentre)
Ray Borrow (Manchester Public Health Laboratory)
John McLeod Griffiss (University of California)
Francis Bajunirwe (MUST)
Theresa Aguado (WHO)
Lisbeth Meyer Næss (NIPH)

Data Safety Monitoring Board:

Dr Ambrose Talisuna, Epidemiology and Surveillance department, Ministry of Health, Uganda

Dr Rosamund Lewis, Immunisation Advisor, WHO, Uganda

CONTENT

Background 7

Hypothesis 10

Objectives 11

Main objective 11

Primary objectives 11

Secondary objectives 11

Investigation plan 12

Study area and study population 12

Study design 13

Vaccines 13

Recruitment of the participants 14

Randomization 14

Blind procedures 15

Sample size in each group 15

Eligibility criteria 16

Adverse Events (AE) and Serious Adverse Events (SAE) 17

Serology 21

Quality control 22

Carriage study 23

Statistical analysis 23

Study duration 24

Regulatory and ethical considerations 24

Regulatory Authority Approval 24

Ethical Conduct of the Study and Ethics Approval 25

Expected benefits, risks and inconveniences 25

Incentive to the study 26

Data Monitoring and Safety Committee 26

Informed consent 26

Collaborative Institutions 27

References 30

Appendix 1 : Economic analysis of potential benefit of reduced dose vaccine 34

Appendix 2 : Declaration of Helsinki 37

Appendix 3 : Menomune® – Aventis Pasteur technical form 40

Appendix 4 : Estimated Ugandan Population aged 2 to 20 (2002/03) 47

Appendix 5 : Informed consent statement 48

Appendix 6 : Case Report Form (CRF) 53

Project collaborators

Project title: Assay of the immunogenicity of reduced dose tetravalent A, C, Y, W135 meningococcal polysaccharide vaccine in Africa.

Principal Investigator: **Philippe J. Guérin,** MD, MPH

Epicentre

8 rue Saint Sabin

75011 Paris

Tel. +33.(0).1.40.21.28.48

E-mail: philippe.guerin@epicentre.msf.org

Co-investigators:

Norwegian Institute of Public Health (NIPH), Division of Infectious Disease Control

PO Box 4404, Nydalen, NO-0403 Oslo, Norway

Dominique A. Caugant, PhD (Co-Principal Investigator)

WHO Collaborating Centre for Reference and Research on Meningococci

Ingeborg Aaberge, MD, PhD

Einar Rosenqvist, PhD

E. A. Høiby, MD

John-Arne Røttingen MD, PhD

Oddvar Frøholm MD, PhD

Lisbeth Meyer Næss MSc, PhD

Manchester Public Health Laboratory

Withington Hospital, Nell Lane, West Didsbury, Manchester, M20 2LR, United Kingdom

Ray Borrow PhD

Head of Vaccine Evaluation Department

University of California

San Francisco, California 94121

John McLeod Griffiss PhD

Centre for Immunochemistry and Department of Laboratory Medicine

Mbarara University of Science and Technology

P.O Box 1410 Mbarara

Francis Bajunirwe MD, MPH

Epicentre Uganda Research Base

16, Kiwafu Crescent Kansanga. P.O. Box 2362 Kampala

Laurence Ahoua MD, MPH (Co-Principal Investigator)

Patrice Piola MD, MPH

Carole Fogg MPH

Eugene Ruzagira MD

Joseph Babigumira MD

Epicentre Paris

8 Rue Saint Sabin, 75011 Paris, France

Nicolas Nathan MD, MPH

Lorextu Pinoges MSc

Background

Although meningococcal meningitis can occur anywhere in the world, the largest epidemics occur in Africa, in an area known as the "meningitis belt". This belt includes 18 sub-Saharan countries from The Gambia, in the west, to Ethiopia, in the east. Until this year, the majority of outbreaks in that region has been caused by *Neisseria meningitidis* serogroup A along with a smaller contribution of serogroup C [1]. Major epidemics have occurred in the meningitis belt every 5 to 10 years since the beginning of the 20th century [2]. The cycles of outbreaks have, however, been less obvious in the last decades, hence making it more difficult to predict their occurrence. In 1996, the largest meningococcal epidemic so far affected the meningitis belt, resulting in 200,000 reported cases and 20,000 deaths (WHO figures, substantially underestimated) [3].

N. meningitidis serogroup W135 (W135) was first described in 1968. Cases caused by this capsular polysaccharide type have since been reported sporadically worldwide. This serogroup was, until now, not contributing significantly to epidemics and therefore was considered of little epidemiological importance. A limited number of *N. meningitidis* serogroup W135 cases have been confirmed in Africa since the early 80's [4,5]. In 1994, Kwara and coll. reported cases of meningitis caused by serogroup W135 in Mali (during a serogroup A epidemic) and in 1995, such strains were found in The Gambia [6]. Major concerns arose when a W135 strain was identified from cases in a large outbreak among Hajj pilgrims coming back from Mecca, Saudi Arabia, in 2000 [7]. Cases of W135 meningococcal disease were subsequently reported in Europe [8,9], the United States [10] and from African countries in the meningitis belt [11]. The fear of a worldwide spread of this strain was confirmed when an increased number of W135 cases were reported in 2001, towards the end of the epidemics in Burkina Faso, Central African Republic and Niger [1,12]. A recent increase of meningococcal meningitis caused by W135 has also been reported in Cameroon [5].

In the beginning of 2002, a large meningococcal outbreak took place in Burkina Faso and *N. meningitidis* serogroup W135 was reported to be the causative organism of this outbreak by the National Laboratory in Ouagadougou and the World Health Organisation (WHO) Collaborating Centre For Reference and Research on Meningococci in Oslo, Norway, in April 2002 [13]. The high case fatality ratio in cases infected with *N. meningitidis* serogroup W135 disease compared to other serogroups have raised concern internationally [9].

Rational

Epidemiological surveillance and epidemic incidence thresholds have been used for early detection of meningococcal meningitis epidemics. Most outbreaks so far have been caused by either *N. meningitidis* serogroup A or *N. meningitidis* serogroup C. In outbreak situations, the WHO recommends treating meningitis cases with a single dose of intramuscular oily chloramphenicol [14,15] and initiating a reactive mass vaccination campaigns with the meningococcal A+C polysaccharide vaccine for the entire population from 2 to 30 years of age. Questions have been raised about the current strategy of mass vaccination and its cost-effectiveness, and debates are still ongoing [16-18].

Mass vaccination of the population at risk in Burkina Faso was implemented with the meningococcal A+C polysaccharide vaccine until April 2002, but was stopped when serogroup W135 was shown to be the main capsular type involved.

Because of the global shortage in supply of the tetravalent (A/C/Y/W135) vaccine that would have been needed as part of the epidemic response, the crisis committee convened by the Ministry of Health in Burkina Faso focused the control strategy on enhanced surveillance and efficient case management.

Different factors explain this decision:

- There are no monovalent W135 or divalent A/W PS vaccines licensed to drug regulatory authorities
- Until 2002, the only available vaccine offering a protection against W135 is a tetravalent polysaccharide vaccine A/C/Y/W135 produced by two companies, Aventis Pasteur and GlaxoSmithKline (GSK). The current production capacity remains limited and was not sufficient to cover the demand in Burkina Faso.
- The WHO has negotiated with GSK the production of a trivalent A/C/W135 vaccine for a cost of US$1 per dose. GSK has produced up to 3 million doses for the year 2003. GSK could scale up its production up to 5 million doses for the year 2004 if commitments of potential buyers are made. However, this type of commitments is difficult to secure and the level of production is not ensured for the coming years. In addition price of the vaccine might increase.

- The market price of tetravalent polysaccharide vaccine A/C/Y/W135 varies up to $US 50-55 per dose in industrialised countries. Even if available, the cost per vial would be unaffordable for most developing countries.

On middle term/long term perspective, several pharmaceutical industries are developing a meningococcal tetravalent conjugate vaccine A/C/Y/W135 [19]. However, this tetravalent conjugate vaccine is not expected to be on the market before 5 to 10 years and will probably be even less affordable for developing countries. Only a meningococcal serogroup C conjugate vaccine is currently on the market for £17.95 per dose [20]. The Meningitis Vaccine Project (MVP), a partnership between the Program for Appropriate Technology in Health (PATH) and the WHO is currently exploring possibilities to develop a meningococcal conjugate vaccine against serogroup A. This monovalent conjugate A vaccine is expected to be available for the 2007-2008 epidemic season at an affordable price for developing countries [21].

The current dose used in the licensed tetravalent A/C/Y/W135 polysaccharide vaccine is 50µg of each polysaccharide component. During the 1980's, researchers from the Walter Reed Army Institute of Research (WRAIR) did extensive works on the immunogenicity of meningococcal polysaccharide vaccines in adults. A first study performed by Griffiss, Brandt and coll. reported that doses of 5 µg of group Y and group W 135 polysaccharides were as effective as doses of 50µg in inducing production of bactericidal antibody amounts correlating with functional immunity [22]. A second study concluded that doses of 7.5µg (Y and W) and 15µg (A and C) were sufficient to induce equivalent binding and bactericidal antibody responses as 50µg [23]. Similar conclusion was reported in a third trial [24].

In a more recent study from Granoff et al., 1/50 (1 mcg) of the ordinary dose of tetravalent A/C/Y/W135 vaccine was given [25]. The antibody responses to A and C have been measured, and this low dose was sufficient to mount a C response in most of the subjects, but the dose was less effective in eliciting a response to A. The antibody responses to W135 and Y were not reported.

This approach already has a successful precedent as shown by the work of O. Levine and his colleagues with an Hib conjugate vaccine in Central America: Similar functional antibody activities was elicited using one-half or one-third dose of the vaccine [26-28].

Hypothesis

Lower doses of each A/C/Y/W135 component of the meningococcal polysaccharide vaccine could confer a similar functional immunogenic response as the dose of 50µg currently being used, and subsequently be equally protective.

It would potentially bring two major benefits. Firstly, it would increase the number of tetravalent vaccine doses available on the market. Secondly, it would decrease the cost of the individual vaccine dose (see economic analysis "Appendix 1"). As a result, more people could be vaccinated, and thereby protected against the disease, and to a lower price.

Results obtained with the study on the tetravalent A/C/Y/W135 polysaccharide vaccine would be valid for the trivalent A/C/W135 polysaccharide vaccine.

Objectives

Main objective

To evaluate the use of reduced dose tetravalent meningococcal polysaccharide vaccine to control outbreak caused by *N. meningitidis* serogroup W135

Primary objectives

- To measure the immunogenicity of a dose corresponding to one fifth of the amount of the licensed meningococcal A/C/Y/W135 polysaccharide vaccine, i.e. 10µg for each component
- To measure the immunogenicity of a dose corresponding to one tenth of the licensed meningococcal A/C/Y/W135 polysaccharide vaccine, i.e. 5µg for each component

Secondary objectives

- To determine the pharyngeal carriage of *N. meningitidis* and in particular W135 strains in the study population.

- To determine the natural immunity towards *N. meningitidis* serogroup A, C, Y and W135 before immunisation in the study population.

- To measure a possible waning of immunity at one year and at two years after immunisation.

- To measure the immune response after challenging with a second dose of the commercialised meningococcal A/C/Y/W135 polysaccharide vaccine after one year, in a group of volunteers who have received a reduced dose in day 0.

- To create a network of institutions (NIPH, Ugandan MOH, Mbarara University, WHO, Epicentre, MSF) able to co-ordinate efforts and to give a more appropriate response for later outbreaks.

- To strengthen local research capacities through the training of local researchers and technicians.

Investigation plan

Epicentre is the promoter of this clinical trial and will assure the co-ordination of the study with the partners.

Study area and study population

The study must be conducted in a population in Africa that has not been exposed to outbreaks of W135, but is a population at risk of meningococcal meningitis epidemics. Mbarara District was chosen, principally because this area has not experienced a recent epidemic of meningococcal meningitis, a factor that excludes the presence of a high level of background antibodies from previous infections. Furthermore, neighbouring countries, such as Burundi and Rwanda, have faced in 2002 and 2003 respectively outbreaks of meningococcal meningitis serogroup A forcing health authorities to vaccinate massively at risk population [29,30]. By its geographical situation, possible outbreaks of the same etiological pathogen is a permanent threat for Uganda, and more specifically for the Mbarara region. Mbarara is also a research base for the epidemiology agency Epicentre in Uganda. Thus it provides highly competent human resources and logistic capacity essential for such a study.

Within the district of Mbarara, Rwampara County was chosen as the site for recruitment of study participants. Stability of the study population was assessed before choosing the site of immunization. The district directorate of health services for Mbarara District provided advise in the selection of this site. Rwampara County is located 15 km southwest of Mbarara Town, on the Mbarara-Kabale Highway. The county is mostly rural with scattered trading centers and population in Rwampara is predominantly subsistence farmers.

The main health unit in the county or health sub district is the Kinoni Health Center IV. There is a medical officer in charge of the health centre and a team of nurses and support staff assists him. The health sub district is also a suitable site for this interventional study because it has had long standing collaboration with Mbarara University, department of Community Health. The area is participating in mosquito and child health projects both coordinated by Mbarara University. There is also a community based health care (CBHC) program where lay persons are trained as facilitators of health activities in the community. This infrastructure will be utilized in the follow up of study volunteers.

Study design

The study design is a randomised single-blind controlled trial.

Vaccines

The vaccine doses to be tested are based on the reports of Mc Griffiss et al.

First injection

Three groups will be used in this clinical trial:

- **Group 1** will receive a dose of 50µg of each component of A/C/Y/W135 polysaccharide vaccine (currently used dose = 0.5 ml)
- **Group 2** will receive a 1/5 volume (0.1 ml) of the tetravalent vaccine (10µg of each component)
- **Group 3** will receive a 1/10 volume (0.05 ml) of the tetravalent vaccine (5µg of each component of meningococcal A/C/Y/W 135 polysaccharide vaccine)

Volunteers will receive randomly one of the 3 doses of vaccine.

Second injection: Challenge with a second dose of the commercialised meningococcal A/C/Y/W135 polysaccharide vaccine

Widespread use of meningococcal A and C polysaccharide vaccines has raised concerns about inductions of hypo-responsiveness to these polysaccharides [31-33]. In order to assess this parameter, a random cluster of 40 volunteers from the group 1, 2 and 3 will be given a second injection with 50µg of each component of A/C/Y/W135 polysaccharide vaccine one year after the first injection.

Table 1 : Dose injected per group and number of volunteers recruited (n=720)

	**First injection Day 0**		**Second injection after one year**	
	**Dose**	**Number of volunteers**	**Dose**	**Number of volunteers**
**Group 1**	50µg	280	50µg	40
**Group 2**	10µg	220	50µg	40
**Group 3**	5µg	220	50µg	40
**Total**	**-**	**720**	**-**	**120**

Injection of the vaccines

The vaccines use for the study are manufactured by Aventis Pasteur Inc., commercialised as Menomune®.

The vaccine will be injected subcutaneously.

Low volume syringes will be used to inject lower doses of vaccine.

- 0.80 ml for the 50µg dose
- 0.16 ml for the 10µg dose
- 0.08 ml for the 5µg dose (Luer syringe B/BRAUN Omnifix-F®)

These dosages are based on the availability for the study of monodose A/C/Y/W135 polysaccharide vaccine currently on the market.

<u>Recruitment of the participants</u>

Stability of the study population will be assessed before choosing the site of immunization.

In collaboration with the Mbarara health authorities and the Mbarara University Teaching Hospital, the recruitment of the participants for the clinical trial will be done on a volunteer basis.

Volunteers will be recruited in the age group from 2 to 20 years old. The age distribution of the volunteers will be matched to the Ugandan age distribution of the 2-20 years old extracted from the "2002/03 Uganda National Household Survey" (see Appendix 3).

Before inclusion, a parental agreement as well as an informed signed consent, are mandatory for all participants (*see regulatory and ethical considerations*).

An interview will be performed for each volunteer (children, parents or guardians) included in the study. The questionnaire will collect medical history information and vaccination history. Confidential information (e.g name, contact home address) will be separately recorded and kept disjointedly from the Case report form, in order to respect privacy and confidentiality for participants.

<u>Randomization</u>

Once the informed consent signed and clinical examination done, each participant will be randomly allocated to one of the 6 trial groups using a bloc randomization method stratified by age groups. The 6 trial groups are built according to the dosages of vaccine (group 1, 2 and 3) and the immunization regimen (subgroup a or b):

- group 1a (n= 240) : single injection at Day 0 of 50µg of each component;
- group 1b (n= 40) : first injection at Day 0 of 50µg of each component and second injection after one year of 50µg of each component;
- group 2a (n=180) : single injection at Day 0 of 10µg of each component;
- group 2b (n=40) : first injection at Day 0 of 10µg of each component and second injection after one year of 50µg of each component;
- group 3a (n=180) : single injection at Day 0 of 5µg of each component;
- group 3b (n=40) : first injection at Day 0 of 5µg of each component and second injection after one year of 50µg of each component;

Blind procedures

The study is a single-blind randomized controlled clinical trial. After randomization, participants will receive one of the 3 doses of vaccine described previously. The injection will be made without the participant knowing which dosage of vaccine he/she will receive. In addition, the immunogenicity response assay carried out at NIPH will be performed blinded, that is without the laboratory knowing the group to which the participants belong until completion of the analyses.

Sample size in each group

The study is a non inferiority trial to prove that the reduced doses of the vaccine elicit equivalence responses to the currently licensed vaccine dose.

According to the literature, individual responses to all four polysaccharide antigens as determined by serum bactericidal assays (SBA) [defined as fourfold or greater increase between pre and postsera antibody after immunisation] is expected to be over 90% [34-37]. These studies have been performed in healthy adult volunteers in USA.

In the study we plan to conduct in Uganda, presenting a population of young children and potential subclinical nutrition deficiencies among the volunteers, the antibody responses to immunisation with the meningococcal A/C/Y/W135 polysaccharide vaccine is expected to be lower. We have used an estimate of 80% for the calculation of the sample size. Assuming a higher proportion of responders will lead to lower sample sizes but will be less realistic.

The needed sample size in this study has been calculated by choosing a significance level (one sided) of α of 5% and power of 80%. We expect equal proportions of responders (above a cut off on the protective antibody levels) in all groups given the vaccine being 80%. We have decided to accept a difference limit Δ of 10%. This gives a required sample size of 198 persons in each

group. Because the reference group (license vaccine) will be used for 2 comparisons, a correction of ($N = n \sqrt{\text{no of comparisons}}$) was applied (Lellouch & Lazar, 1974), bringing that group to 280. The calculations have been performed with nQuery Advisor® software.

Taking into account the problems relative to the interpretation of non inferiority studies, particular attention will be given to ensure a good quality follow up of all volunteers. We plan to recruit 280 volunteers in the reference group and 220 in the two other groups – to allow for loses to follow up – a total of 720 participants.

Eligibility criteria

- Inclusion criteria
  - Volunteers should not be suffering of severe chronic disease or a known congenital or acquired immunodeficiency. A medical exam will be performed by a medical doctor before inclusion.
  - Volunteers must be living in Mbarara district and within 15 Km from the site of immunization. Volunteers should be residents of the chosen site and should express no plan of moving from this area during the study period.
  - Volunteers must be available for follow-up for the duration of the study (minimum of 24 months).
  - Able and willing to provide information so that the participant may be located.
- Exclusion criteria
  - Volunteers with severe chronic disease or with a general condition requiring hospital admission.
  - Volunteers with a known congenital or acquired immunodeficiency (e.g. HIV). Diagnosis will be presumptive based on the medical background and the clinical examination. No serological HIV testing will be performed.
  - Evidence of any concomitant infection at the time of presentation (including rashes other than scabies, ear, nose or throat infections, and abnormal respiratory system examination).
  - The patient has any other underlying disease that compromise the diagnosis and the evaluation of the response to the study medication.
  - History of serious adverse reactions to vaccines such as anaphylaxis or related symptoms such as hives, respiratory difficulty, angioedema and abdominal pain.

- Malnutrition: The nutritional assessment of children aged 24–59 months, a weight-for-height (W/H) index will be calculated. This index is expressed in standard deviations of a normalised distribution of a reference population [38] (National Centre for Health Statistics, USA). Children under 5 years old with a Z-score inferior to -2 will be excluded. For children over 5 or adults, the clinical examination will be considered.
- Pregnant women and lactating women are not eligible for this trial. All women of child-bearing age must provide a urine sample for pregnancy testing before inclusion and, for sub-group "b", before the second vaccine injection.

NOTE : For the subgroup "b", if a women of child-bearing age becomes pregnant during the first year of follow-up, she will not receive a second injection and will be excluded from the second year analysis.

<u>Adverse Events (AE) and Serious Adverse Events (SAE)</u>

The investigator is responsible for the detection and documentation of events meeting the criteria and definition of an AE or SAE as provided in this protocol. Clinical officers identified for the study, will be under the responsibility of the investigator and be in charge of clinical examination and the assessment and follow-up of AE and SAE.

Definition of an AE

Any untoward medical occurrence in a patient or clinical investigation subject, temporally associated with the use of a medicinal product, whether or not considered related to the medicinal product.

An AE can therefore be any unfavorable and unintended sign (including an abnormal laboratory finding), symptom, or disease (new or exacerbated) temporally associated with the use of a medicinal product. For marketed medicinal products, this also includes failure to produce expected benefits (i.e. lack of efficacy), abuse or misuse.

Examples of an AE **includes**:

- Pain at the site of injection
- Tenderness at the site of injection
- Erythema at the site of injection
- Induration at the site f injection
- Transient headaches

- Transient malaise
- Transient temperature > 37,5 °C (or 100°F)
- Transient chills

Examples of an AE **does not include** a/an:

- Medical or surgical procedure (e.g., endoscopy, appendectomy); the condition that leads to the procedure is an AE.
- Situations where an untoward medical occurrence did not occur (social and/or convenience admission to a hospital).
- Anticipated day-to-day fluctuations of pre-existing disease(s) or condition(s) present or detected at the start of the study that do not worsen.

Definition of a SAE

A serious adverse event is any untoward medical occurrence that, at any dose:

a) results in death.

b) is life-threatening.

NOTE: The term 'life-threatening' in the definition of 'serious' refers to an event in which the subject was at risk of death at the time of the event. It does not refer to an event, which hypothetically might have caused death, if it were more severe.

c) requires hospitalisation or prolongation of existing hospitalisation.

NOTE: In general, hospitalisation signifies that the subject has been detained (usually involving at least an overnight stay) at the hospital or emergency ward for observation and/or treatment that would not have been appropriate in the physician's office or out-patient setting. Complications that occur during hospitalisation are AEs. If a complication prolongs hospitalisation or fulfils any other serious criteria, the event is serious. When in doubt as to whether "hospitalisation" occurred or was necessary, the AE should be considered serious.

Hospitalisation for elective treatment of a pre-existing condition that did not worsen from baseline is not considered an AE.

d) results in disability/incapacity, or

NOTE: The term disability means a substantial disruption of a person's ability to conduct normal life functions. This definition is not intended to include experiences of relatively minor medical significance such as uncomplicated headache, nausea, vomiting, diarrhoea, influenza, and accidental trauma (e.g. sprained ankle) which may interfere or prevent everyday life functions but do not constitute a substantial disruption.

e) is a congenital anomaly/birth defect.

f) Medical or scientific judgement should be exercised in deciding whether reporting is appropriate in other situations, such as important medical events that may not be immediately life-threatening or result in death or hospitalisation but may jeopardize the subject or may require medical or surgical intervention to prevent one of the other outcomes listed in the above definition. These should also be considered serious. Examples of such events are invasive or malignant cancers, intensive treatment in an emergency room or at home for allergic bronchospasm, blood dyscrasias or convulsions that do not result in hospitalisation, or development of drug dependency or drug abuse.

Time Period, Frequency, and Method of Detecting AEs and SAEs

AEs and SAEs will be collected by Medical Officers or Clinical Officers, from the time of informed consent to the time the patient completes the study (day 30 after injection, or withdraw). At Day 0, participants will be observed for at least 1 hour after the injection (similar surveillance will be implemented for the second injection at 12 months for subgroups “b”).

From thereon, assessment of AEs and SAEs will be done on a weekly based consultation in the month following the day of the vaccine injection. Subjects will be ask to return to the immunization site with tracers hired for volunteers not presenting for follow-up.

Apart from scheduled visits for follow-up, participants can come to the Epicentre clinic located at Mbarara University Teaching Hospital for clinical examination whenever necessary.

Adverse events or reactions not previously documented in the study will be recorded in the adverse experience section of the patient's case record form (CRF). The nature of each experience, date and time (where appropriate) of onset, duration, severity and relationship to he injection should be established.

Adverse events or reactions already documented in the CRF i.e. at a previous assessment and designated as 'continuing' should be reviewed. If these have resolved, the documentation in the CRF should be completed.

NB. If an adverse experience changes in frequency or severity during a study period, a new record of the experience will be started.

Ask the patients a non-leading question such as:

"Have you appeared or felt different in any way since starting the new treatment / since the last assessment?”

Recording of AEs and SAEs

When an AE/SAE occurs, it is the responsibility of the investigator to review all documentation (e.g., hospital progress notes, laboratory, and diagnostics reports) relative to the event. The investigator will then record all relevant information regarding an AE/SAE on the CRF.

The investigator will attempt to establish a diagnosis of the event based on signs, symptoms, and/or other clinical information. In such cases, the diagnosis should be documented as the AE/SAE and not the individual signs/symptoms.

Evaluating AEs and SAEs

Assessment of Intensity of AEs and SAEs

The investigator will make an assessment of intensity for each AE and SAE reported during the study. The assessment will be based on the investigator's clinical judgement. The intensity of each AE and SAE recorded in the CRF should be assigned to one of the following categories:

- Mild: An event that is easily tolerated by the subject, causing minimal discomfort and not interfering with everyday activities.
- Moderate: An event that is sufficiently discomforting to interfere with normal everyday activities.
- Severe: An event that prevents normal everyday activities.

An AE that is assessed as severe should not be confused with a SAE. Severity is a category utilised for rating the intensity of an event; and both AEs and SAEs can be assessed as severe.

Assessment of Causality of AEs and SAEs

The investigator is obligated to assess the relationship between investigational product and the occurrence of each AE/SAE. The investigator will use clinical judgement to determine the relationship. Alternative causes, such as natural history of the underlying diseases, concomitant therapy, other risk factors, and the temporal relationship of the event to the investigational product will be considered and investigated. The investigator will also consult the CIB/IB and/or Product Information, for marketed products, in the determination of his/her assessment.

The investigator will provide the assessment of causality as per instructions on the SAE form in the CRF.

Follow-up of AEs and SAEs

After the initial AE/SAE report, the investigator is required to proactively follow each subject and provide further information on the subject's condition.
All AEs and SAEs documented at a previous visit/contact and are designated as ongoing, will be reviewed at subsequent visits/contacts.
All AEs and SAEs will be followed until resolution, until the condition stabilises, until the event is otherwise explained, or until the subject is lost to follow-up. Once resolved, the appropriate AE/SAE CRF page(s) will be updated. The investigator will ensure that follow-up includes any supplemental investigations as may be indicated to elucidate the nature and/or causality of the AE or SAE. This may include additional laboratory tests or investigations, histopathological examinations, or consultation with other specific health care professionals.

Serology

Serum samples will be collected from each volunteer at the time of vaccination and at 4 weeks, 12 months (cluster group of 40 persons in each group 1,2 and 3), 13 months (cluster group of 40 persons in each group 1,2 and 3), and 24 months after vaccination. We allow a delay of more or less 10 days with regard to the planned date of blood collection.
Ten ml of whole blood will be collected for antibody determinations.

The immune responses to the different doses of the A/C/Y/W135 vaccine will be assayed with enzyme linked immunosorbent assay (ELISA) and serum bactericidal activity (SBA). In ELISA IgG antibodies to each separate polysaccharide A, C, Y and W135 will be measured by a standard method [39,40]. In addition, in a subset of sera (10% of the samples) will be tested using an ELISA method measuring higher-avidity antibodies [41]. The SBA assays for each serogroup will be based on the NIPH experience with measuring SBA against serogroup B [42,43] and modified according to Borrow et al.[44]. The SBA assay will be performed with the tilt method (reaction mixture after incubation is plated onto agar plates to count surviving colony forming units (CFUs)) applying baby rabbit complement [44,45]. When establishing the SBA, human complement will also be used in a subset of sera (10% of the samples) for comparison [46,47]. The importance of complement source will initially be tested with both human and rabbit complement in pre and post vaccination sera (4 weeks) from 25% of the vaccinees from group 1a (= 60) sera from

individuals who have got 50 mcg (covering all age groups). Depending of the results from this analysis, we may eventually come back and study other groups with human complement. Standardised inocula for each serogroup of organism examined will be used. A four-fold increase in the SBA assay titer is chosen as a criterion for significant increase in bactericidal antibodies. In addition, geometric mean titers will also be calculated. As methods for serogroups W 135 and Y in particular are not validated, modifications of the methods may be used in addition to the primary methods. The ELISA and SBA assays will be performed at NIPH. Emphasis will be put on analysing the response to W 135 first.

Table 2 : Schedule of serology testing per test and number of subjects tested

			Day 0		4 weeks		12 months		13 months		2 years	
	Sub-group*	n	ELISA 1	SBA 1	ELISA 2	SBA 2	ELISA 3	SBA 3	ELISA 4	SBA 4	ELISA 5	SBA 5
Group 1 (n=280)	1a	240	X	X	X	X					X	X
	1b	40	X	X	X	X	X	X	X	X	X	X
Group 2 (n=220)	2a	180	X	X	X	X					X	X
	2b	40	X	X	X	X	X	X	X	X	X	X
Group 3 (n=220)	3a	180	X	X	X	X					X	X
	3b	40	X	X	X	X	X	X	X	X	X	X
Total		720	720	720	720	720	120	120	120	120	720	720

NB: Taking into account the public health priorities and the logistic constraints, ELISA and SBA testing will be performed on serogroup A and W135 in order to allow publications of the results of ELISA 1&2 and SBA 1&2 in the six months following the beginning of the clinical trial. ELISA and SBA testing of the serogroup C and Y will be performed later. Sera for ELISA 3 and SBA 3 will be collected the same day before the second injection in the subgroups b. Sera for ELISA 4 and SBA 4 will be collected 4 weeks after the second injection in the sub-groups b.

Quality control

To provide confirmatory testing as a quality control, a collaboration has been established with Manchester Public Health Laboratory, in the UK. This laboratory will analyse about 10% of the samples from day zero and day 4 weeks.

Carriage study

Posterior pharyngeal samples will be collected from the volunteers at 0 and 4 weeks. Samples will be plated directly on selective (VCN) chocolate agar and forwarded immediately to the laboratory in the country where the study is performed. Plates will be incubated for 2 days. Meningococci will be identified by standard laboratory methods [48]. One colony from each throat culture will be subcultured twice and preserved frozen for further analyses. The serogroup will be determined by agglutination with commercial antisera. Preserved isolates will be forwarded to the NIPH, Oslo for further characterization using monoclonal antibodies and molecular techniques.

Volunteers who will be found carriers of *N. meningitidis* of a homologous serogroup at any time will be excluded from the analysis of response to that polysaccharide.

Statistical analysis

The definition of a adequate responder is any participant with a four-fold increase in the SBA assay titer.

The baseline characteristics of the participants of each experimental arm will be presented using descriptive statistics and compared by Chi2 or Student's t-test analyses.

Results of the carriage study will be presented using descriptive statistics. Numbers of carriers of each homologous serogoup of N. meningitis will be presented.

The proportion of adequate responses in each group will be expressed as a percentage with associated 95% confidence intervals (response to vaccine rate). Results will be primarily based on a per-protocol analysis, however an intention-to-treat (ITT) analysis will also be performed.

Results for vaccine efficacy will be based on SBA and used to test for non-inferiority.

Safety and tolerability analysis will be conducted on all patients who received at least one vaccine injection. Adverse Events will be listed in order of frequency, and described according to median duration, severity, and likelihood that they were related to the study vaccine. The incidence of Adverse Events in the three groups will be estimated. Any Severe Adverse Events will be described in detail. Outcomes of Adverse Events and Serious Adverse Events (recovery, sequelae, etc.) will also be presented.

Study duration

The total duration of the study is 36 months.

- *First analysis: 6 months*

Results of the carriage study and the antibody determinations (serology at 0 and 4 weeks) are expected to be available after 6 months. Taking into account the public health priorities and the logistic constraints, ELISA and SBA testing will be performed on serogroup A and W135 in order to allow publications of the results of ELISA 1 & 2 and SBA 1 & 2 in the six months following the second blood sample (ELISA 2 & SBA 2). ELISA and SBA testing of the serogroup C and Y will be performed later.

- *Second analysis: 18 months*

  Results the antibody determinations (serology at 12 months and 13 months) are expected to be available after 18 months.

- *Third analysis:*

  Results the antibody determinations (serology at 24 months) are expected to be available after 30 months.

- *Intermediate analysis:*

  An intermediate analysis will be performed at the end of the phase 1 study period. At this stage, if the hypothesis tested appears not to be valid, the phase 2 and 3 study will be aborted, after approval of the scientific committee.

Regulatory and ethical considerations

Regulatory Authority Approval

Epicentre, NIPH will obtain approval to conduct the study from the Mbarara University of Science and Technology (MUST) Faculty Research and Ethics Committee, from the MUST Institutional Ethics Committee and from the Uganda National Committee of Science and Technology.

The protocol must also receive official backup from the Epidemiology and Surveillance department of the Uganda Ministry of Health.

The protocol will also be presented to the National Committee for Medical Research Ethics in Norway. The same standards apply for the conduct of this study in the hosting country as for an equivalent study in Norway. The study will start only when all the ethical approvals have been obtained.

Ethical Conduct of the Study and Ethics Approval

This study will be conducted in accordance with "good clinical practice" (GCP) and all applicable regulatory requirements, including, where applicable, the 1996 version of the Declaration of Helsinki (Appendix 2).

The investigator (or sponsor, where applicable) is responsible for ensuring that this protocol, the site's informed consent form, and any other information that will be presented to potential subjects (e.g., advertisements or information that supports or supplements the informed consent) are reviewed and approved by the appropriate IEC/IRB. The investigator agrees to allow the IEC/IRB direct access to all relevant documents. The IEC/IRB must be constituted in accordance with all applicable regulatory requirements.

If the protocol, the informed consent form, or any other information that the IEC/IRB has approved for presentation to potential subjects is amended during the study, the investigator is responsible for ensuring the IEC/IRB reviews and approves, where applicable, these amended documents. The investigator must follow all applicable regulatory requirements pertaining to the use of an amended informed consent form including obtaining IEC/IRB approval of the amended form before new subjects consent to take part in the study using this version of the form.

Expected benefits, risks and inconveniences

Uganda, more specifically the South-West region including Mbarara district, is at risk of meningitis outbreaks. Indeed, neighbouring countries have recently experienced epidemics of meningococcal meningitis. By participating in the study, subjects will be protected against all 4 serogroups of *N. meningitides* if such an outbreak occurs.

The study vaccine, Menomune®- A/C/Y/W-135 , is commercialised for more than 20 years in the United States, Canada and Europe. Adverse events are well documented and rare (see Appendix 3). Risks and inconveniences for the participants should be minor in the study. Nevertheless, precautions will be taken by the investigators for the safe and effective use of the vaccine (e.g.

Epinephrine injection will be immediately available to treat unexpected anaphylactic or other allergic reactions).

Incentive to the study

Incentive will systematically be proposed to all participants of the study: impregnated mosquito nets and transport refund will be provided when necessary.

Data Monitoring and Safety Committee

A Data Monitoring and Safety Committee will be created, including a staff member of the immunization department of the WHO and a person from the Epidemiology and Surveillance department of the Ugandan Ministry of Health. The Committee will be in charge of verifying the quality of the databases used for analysis. If a Meningococcal meningitis epidemic occurs during the study, measures based on the international recommendations will be taken in collaboration with the Ministry of Health for prevention and case treatment [49]. In this case, the Committee will reserve itself the right to evaluate the need for a “rescue” immunization for participants who have received fractional doses of the A/C/Y/W135 vaccine according to the period of the outbreak, the point in time of the study and the age group to be vaccinated.

Informed consent

Informed consent will be obtained before the subject can participate in the study. The contents and process of obtaining informed consent will be in accordance with all applicable regulatory requirements.

Information should be given in both oral and written form, in presence of an eye witness, whenever possible and deemed appropriate by the ERC/IRB. Only subjects giving their authorisation and signing an informed consent will be enrolled. For all participants, parents or guardians will be approached for consent.

Consent forms will be translated into the local language, Runyankore, and must use a vocabulary fully comprehensible to the prospective patient, their relatives, guardians or, if necessary, legal representatives. Informed consent shall be documented by the use of a written consent form approved by the ERC/IRB and signed by the patient or the patient's legally authorised representative.

The written consent document will embody the elements of informed consent as described in the Declaration of Helsinki and will also comply with local regulations. The explanation should include the aim of the study, the expected benefits for the participants, the risks and inconveniences.

Consent must be documented either by the dated signature of the patient or of an independent witness. The signature confirms the consent is based on information that has been read and understood. If the volunteer is unable to write their signature then a thumbprint may be used.

If the volunteer is unable to read the information herself, full and comprehensive information must be communicated to the potential in the presence of a witness. The witness will be an independent third party i.e. not a nominated co-investigator. The witness will sign the informed consent form (testifying that informed consent has been given verbally) along with the investigator (or her nominated representative).

Each patient's signed informed consent form must be kept on file by the investigator for possible inspection by Regulatory Authorities.

Collaborative Institutions

a) The Ugandan Ministry of Health (MOH), at its local and national levels

b) The Mbarara University of Science and Technology

c) Norwegian Institute of Public Health brings technical expertise in meningococci microbiology laboratory, vaccine development and epidemiology

d) Epicentre, Paris, France and the Mbarara research base, Uganda will perform and co-ordinate the field study

e) The Ugandan representation of the WHO, and the regional office (WHO/AFRO)

f) WHO gives the necessary political and technical support and possible financial resources

g) Aventis Pasteur will provide necessary tetravalent polysaccharide vaccines (Menomune®) needed for the clinical trial

h) Manchester Public Health Laboratory in the UK will assure quality control of serology testing

<u>Area of expertise of participative institutions</u>

Norwegian Institute of Public Health, Oslo, Norway (NIPH)

The NIPH is the national public health institute of Norway, founded in 1929. It is under the authority of the Ministry of Health and Social Affairs. The institute is equipped with all basic and technological advanced instrumental tools and facilities for research in epidemiology, vaccinology, bacteriology and immunology. The Division for Infectious Disease Control has long experience with research, production and control of group B meningococcal vaccines and has been a WHO Collaborating Centre for Reference and Research on Meningococci since 1991. NIPH has therefore been doing extensive research on genetic structure of meningococcal populations, meningococcal immunology and antigenic studies of *Neisseria meningitidis* strains and outer membrane vesicle vaccines against serogroup B meningococci. For more information, see http://www.fhi.no

Epicentre

Epicentre is a non-profit organisation created in 1987 by Médecins Sans Frontières, which groups health professionals specialised in public health and epidemiology. In 1996, Epicentre became a World Health Organization Collaborating Centre for Research in Epidemiology and Response to Emerging Diseases. Epicentre's team carries out operational research studies from its bases in Paris, Geneva and Brussels, and a field research base in Uganda. Epicentre also offers its expertise to organisations requesting short field epidemiology missions in developing countries. The main objective of Epicentre’s research is to provide practical answers to the questions of health professionals working in the field. Each project is coordinated by a permanent member of the research team and carried out in collaboration with national and international partners. These exchanges favour the transfer of knowledge and the training of the participants involved.

Research undertaken by Epicentre uses a variety of methods including clinical trials, observational studies (cohort studies, case-control studies), decision analysis, cross-sectional sample surveys and analysis of surveillance data. Principle research areas developed by Epicentre over the last 10 years include research in infectious and parasitic diseases, nutrition, vaccinology and epidemiology of disasters and population displacements.

Other the past 15 years, several projects on bacterial meningitis has been carried out by Epicentre, giving the agency a unique expertise on this subject in developing countries :

- Clinical trials on the therapy of bacterial meningitis in children (Niger and Mali, 1989-1995).
- Modelling transmission of N. meningitidis group A during outbreaks in Sahelian areas and assessment of mass vaccination impact (1997).
- Assessment of alert thresholds for detecting outbreaks of bacterial meningitidis in Africa (1997).
- Oily Chloramphenicol and treatment of meningitis - Literature review (2000).
- Bio-equivalence study on the efficacy of cephtriaxone versus oily chloramphenicol in the treatment of meningococcal meningitidis infections in Mali (2003).

References

1. Epidemics of meningococcal disease. African meningitis belt, 2001. *Wkly.Epidemiol.Rec.* 2001;**76**:282-8.

2. Olyhoek T, Crowe BA, Achtman M. Clonal population structure of Neisseria meningitidis serogroup A isolated from epidemics and pandemics between 1915 and 1983. *Rev.Infect.Dis.* 1987;**9**:665-92.

3. Response to epidemic meningitis in Africa, 1997. *Wkly.Epidemiol.Rec.* 1997;**72**:313-8.

4. Denis F, Rey JL, Amadou A, Saliou P, Prince-David M, M'Boup S *et al*. Emergence of meningococcal meningitis caused by W 135 subgroup in Africa. *Lancet* 1982;**2**:1335-6.

5. Fonkoua MC, Taha MK, Nicolas P, Cunin P, Alonso JM, Bercion R *et al*. Recent increase in meningitis Caused by Neisseria meningitidis serogroups A and W135, Yaounde, Cameroon. *Emerg.Infect.Dis.* 2002;**8**:327-9.

6. Kwara A, Adegbola RA, Corrah PT, Weber M, Achtman M, Morelli G *et al*. Meningitis caused by a serogroup W135 clone of the ET-37 complex of Neisseria meningitidis in West Africa. *Trop.Med.Int.Health* 1998;**3**:742-6.

7. Taha MK, Achtman M, Alonso JM, Greenwood B, Ramsay M, Fox A *et al*. Serogroup W135 meningococcal disease in Hajj pilgrims. *Lancet* 2000;**356**:2159.

8. Matsika-Claquin MD, Perrocheau A, Taha MK, Levy-Bruhl D, Renault P, Alonso JM *et al*. [Meningococcal W135 infection epidemics associated with pilgrimage to Mecca in 2000]. *Presse Med.* 2001;**30**:1529-34.

9. Hahne SJ, Gray SJ, Jean F, Aguilera, Crowcroft NS, Nichols T *et al*. W135 meningococcal disease in England and Wales associated with Hajj 2000 and 2001. *Lancet* 2002;**359**:582-3.

10. From the Centers for Disease Control and Prevention. Risk for meningococcal disease associated with the Hajj 2001. *JAMA* 2001;**285**:1438.

11. Yousuf M,.Nadeem A. Meningococcal infection among pilgrims visiting Madinah Al-Munawarah despite prior A-C vaccination. *J.Pak.Med.Assoc.* 2000;**50**:184-6.

12. Taha MK, Parent dC, I, Schlumberger M, Sanou I, Djibo S, de Chabalier F *et al*. Neisseria meningitidis serogroups W135 and A were equally prevalent among meningitis cases occurring at the end of the 2001 epidemics in Burkina Faso and Niger. *J.Clin.Microbiol.* 2002;**40**:1083-4.

13. Meningococcal disease, serogroup W135, Burkina Faso. *Wkly.Epidemiol.Rec.* 2002;**77**:152-5.

14. Meningococcal meningitis in Africa. *Wkly.Epidemiol.Rec.* 1990;**65**:120-2.

15. World Health Organisation. Control of epidemic meningococcal disease. WHO practical guidelines. 2nd Edition. World Health Organisation. WHO/EMC/BAC/98.3. 1998.

16. Woods CW, Armstrong G, Sackey SO, Tetteh C, Bugri S, Perkins BA *et al*. Emergency vaccination against epidemic meningitis in Ghana: implications for the control of meningococcal disease in West Africa. *Lancet* 2000;**355**:30-3.

17. Lewis R, Nathan N, Communier A, Varaine F, Fermon F, Chabalier FD *et al*. [Detection of meningococcal meningitis epidemics in Africa: a new recommendation]. *Sante* 2001;**11**:251-5.

18. Parent du Chatelet I, Gessner BD, da Silva A. Comparison of cost-effectiveness of preventive and reactive mass immunization campaigns against meningococcal meningitis in West Africa: a theoretical modeling analysis. *Vaccine* 2001;**19**:3420-31.

19. Jodar L, Feavers IM, Salisbury D, Granoff DM. Development of vaccines against meningococcal disease. *Lancet* 2002;**359**:1499-508.

20. Trotter CL,.Edmunds WJ. Modelling cost effectiveness of meningococcal serogroup C conjugate vaccination campaign in England and Wales. *BMJ* 2002;**324**:809.

21. Jodar, L., Laforce, M., and Granoff, D. M. The Meningitis Vaccine Project (MVP): a new model for public/private sector partnerships for development and introduction of new vaccines for prevention of infectious diseases in the developing world. Thirteenth International Pathogenic Neisseria Conference. Norwegian Institute of Public Health,Oslo,Norway; Sept.1-6,2002, p23. 2002.

22. Griffiss JM, Brandt BL, Broud DD. Human immune response to various doses of group Y and W135 meningococcal polysaccharide vaccines. *Infect.Immun.* 1982;**37**:205-8.

23. Griffiss JM, Brandt BL, Broud DD, Altieri PL, Berman SL. Relationship of dose to the reactogenicity and immunogenicity of meningococcal polysaccharide vaccines in adults. *Mil.Med.* 1985;**150**:529-33.

24. Brandt BL, Griffiss JM, Broud DD, Boslego JW. Tetravalent meningococcal polysaccharide vaccine. Relashionship of dose to immunogenicity and reactogenicity in young adults. In Kluwer Academic Publishers, ed. *Gonococci and Meningococci*, pp 33-7. Dordrecht, The Netherlands: 1988.

25. Granoff DM, Gupta RK, Belshe RB, Anderson EL. Induction of immunologic refractoriness in adults by meningococcal C polysaccharide vaccination. *J.Infect.Dis.* 1998;**178**:870-4.

26. Lagos R, Valenzuela MT, Levine OS, Losonsky GA, Erazo A, Wasserman SS *et al*. Economisation of vaccination against Haemophilus influenzae type b: a randomised trial of immunogenicity of fractional-dose and two-dose regimens. *Lancet* 1998;**351**:1472-6.

27. Fernandez J, Balter S, Feris J, Gomez E, Garib Z, Castellanos PL *et al*. Randomized trial of the immunogenicity of fractional dose regimens of PRP-T Haemophilus influenzae type b conjugate vaccine. *Am.J.Trop.Med.Hyg.* 2000;**62**:485-90.

28. Romero-Steiner S, Fernandez J, Biltoft C, Wohl ME, Sanchez J, Feris J *et al*. Functional antibody activity elicited by fractional doses of Haemophilus influenzae type b conjugate vaccine (polyribosylribitol phosphate-tetanus toxoid conjugate). *Clin.Diagn.Lab Immunol.* 2001;**8**:1115-9.

29. Meningococcal disease, Great Lakes area (Burundi, Rwanda, United Republic of Tanzania)--update. *Wkly.Epidemiol.Rec.* 2002;**77**:317.

30. Molesworth AM, Thomson MC, Connor SJ, Cresswell MP, Morse AP, Shears P *et al*. Where is the meningitis belt? Defining an area at risk of epidemic meningitis in Africa. *Trans.R.Soc.Trop.Med.Hyg.* 2002;**96**:242-9.

31. Anderson EL, Bowers T, Mink CM, Kennedy DJ, Belshe RB, Harakeh H *et al*. Safety and immunogenicity of meningococcal A and C polysaccharide conjugate vaccine in adults. *Infect.Immun.* 1994;**62**:3391-5.

32. MacLennan J, Obaro S, Deeks J, Williams D, Pais L, Carlone G *et al*. Immune response to revaccination with meningococcal A and C polysaccharides in Gambian children following repeated immunisation during early childhood. *Vaccine* 1999;**17**:3086-93.

33. Borrow R, Joseph H, Andrews N, Acuna M, Longworth E, Martin S *et al*. Reduced antibody response to revaccination with meningococcal serogroup A polysaccharide vaccine in adults. *Vaccine* 2000;**19**:1129-32.

34. Wong KH, Barrera O, Sutton A, May J, Hochstein DH, Robbins JD *et al*. Standardization and control of meningococcal vaccines, group A and group C polysaccharides. *J.Biol.Stand.* 1977;**5**:197-215.

35. Farquhar JD, Hankins WA, Desanctis AN, Demeio JL, Metzgar DP. Clinical and serological evaluation of purified polysaccharide vaccines prepared from Neisseria meningitidis group Y. *Proc.Soc.Exp.Biol.Med.* 1977;**155**:453-5.

36. Farquhar JD, Hankins WA, Desanctis AN, Demeio JL, Metzgar DP. Clinical and serological evaluation of a meningococcal polysaccharide vaccine groups A, C, and Y. *Proc.Soc.Exp.Biol.Med.* 1978;**157**:79-82.

37. Hankins WA, Gwaltney JM, Jr., Hendley JO, Farquhar JD, Samuelson JS. Clinical and serological evaluation of a meningococcal polysaccharide vaccine groups A, C, Y, and W135. *Proc.Soc.Exp.Biol.Med.* 1982;**169**:54-7.

38. Dibley MJ, Goldsby JB, Staehling NW, Trowbridge FL. Development of normalized curves for the international growth reference: historical and technical considerations. *Am.J.Clin.Nutr.* 1987;**46**:736-48.

39. Gray S.J, Borrow R, Kaczmarski E.B. Meningococcal serology. In A.J.Pollard, M.C.Maiden, eds. *Meningococcal Disease*, pp 61-87. Totowa, New Jersey: Humana Press, 2003.

40. Rosenqvist E, Käyhty H, Pollard A. Determination of Antibody responses to meningococcal antigens by ELlSA. In A.J.Pollard, M.C.Maiden, eds. *Meningococcal Vaccines*, pp 255-73. Totowa, New Jersey: Humana Press, 2003.

41. Granoff DM, Maslanka SE, Carlone GM, Plikaytis BD, Santos GF, Mokatrin A *et al.* A modified enzyme-linked immunosorbent assay for measurement of antibody responses to meningococcal C polysaccharide that correlate with bactericidal responses. *Clin.Diagn.Lab Immunol.* 1998;**5**:479-85.

42. Hoiby EA, Rosenqvist E, Froholm O, Bjune G, Feiring B, Nokleby H *et al.* Bactericidal antibodies after vaccination with the Norwegian meningococcal serogroup B outer membrane vesicle vaccine: a brief survey. *NIPH annal* 1991;**14**:147-56.

43. Rosenqvist E, Hoiby EA, Wedege E, Bryn K, Kolberg J, Klem A *et al.* Human antibody responses to meningococcal outer membrane antigens after three doses of the Norwegian group B meningococcal vaccine. *Infect.Immun.* 1995;**63**:4642-52.

44. Borrow R, Carlone GM. Serogroup B and C serum bactericidal assays. In Pollard AJ MMe, ed. *Meningococcal vaccines: methods and protocols. Methods in molecular medicine*, pp 289-304. Totowa NJ, USA: Humana Press, 2001.

45. Rosenqvist, E., Høiby, EA, Fuglesang, JE, Feiring, B., Aaberge, IS, Jenum, P, Konsmo, K, Forsdahl, B, and Nøkleby, H. Reduced immune response after repeated immunisations with serogroup A+C polysaccharide vaccine in Norwegian teen-agers. 12th International Pathogenic Neisseria Conference . 2003.

46. Norheim, G, Høiby, EA, Caugant, D. A., Namork, E, Tangen, T, Fritzsønn, E, and Rosenqvist, E. Immunogenicity and bactericidal activity in mice of an outer membrane protein vesicle vaccine against Neisseria meningitidis serogroup A disease. Vaccine 2003 (In Press).

47. Norheim, G, Fritzønn, E, Kristiansen, P, Tangen, T, Caugant, D. A., Høiby, EA, Aase, A., Konsmo, K, Aaberge, IS, and Rosenqvist, E. An alternative strategy for vaccines against serogroup A, C, Y and W135 meningococcal disease. 7th EMGM meeting . 24-9-2003.

48. Riou JY, Guibourdenche M. *Neisseria* et *Branhamella.* Methodes de laboratoire. 1992.

49. Detecting meningococcal meningitis epidemics in highly-endemic African countries. *Wkly.Epidemiol.Rec.* 2000;**75**:306-9.

50. Medical department, Médecins Sans Frontières Paris. Cerebro Spinal Meningitis outbreak, Sudan 1999. MSF evaluation. 1999. Unpublished Work report.

Appendix 1 : Economic analysis of potential benefit of reduced dose vaccine

An economic analysis has been conducted in collaboration with Médecins Sans Frontières (MSF), Paris. Economic estimates are based on an MSF cost analysis study of a mass vaccination campaign in the context of a meningitis outbreak in Sudan, 1999 [50].

Method: The analysis included cost of national and international staff, immunisation equipment, vehicles, cold chain, logistics, freight and administrative cost. Cost is given by person vaccinated. MSF used the bivalent A+C meningococcal capsular polysaccharide vaccine (50 doses). As we don't know what will be the packaging used for the trivalent or tetravalent or reduced dose polysaccharide vaccines in developing countries, we assume for this calculation that only the price of the vaccine varies. Even if some difference may be expected in the number of syringes, the cost difference is considered to be negligible compared to other expenses.

The price of the A+C meningococcal vaccine used in the analysis was 0,152 Euro/dose. Based on ICG information, the price of a "full dose" trivalent A/C/W135 vaccine is expected to be at approximately 1 Euro (future vaccine developed by GSK). Cost of the reduced dose vaccine is estimated 20 cents and 10 cents, respectively for 1/5 and 1/10 reduced dose. Calculations are based on the same methodology as the one used by MSF in the initial study.

Similar cost estimates are presented with a tetravalent polysaccharide A,C,Y,W135 vaccine for a price at 3 Euro (price based on estimation given by ICG).

Results 1: Estimated costs of immunisation: bivalent vaccine vs. trivalent vaccines ("full" and reduced dose)

ESTIMATED COST OF IMMUNIZATION

	SUDAN	Vaccine A+C	Vaccine A,C,W	Vaccine A,C,W	Vaccine A,C,W
			Full dose	Reduced dose 1/5	Reduced dose 1/10
**Cost vaccine/dose in EURO**		**0,152**	**1,00**	**0,20**	**0,10**
**Nb of person vaccinated**		**1 916 802**	**1 916 802**	**1 916 802**	**1 916 802**
Nb of days/team		1 271	1 271	1 271	1 271
Cost of standard team/day		106	106	106	106
**Total cost of vacci. teams**		**20 569**	**20 569**	**20 569**	**20 569**
Nb of days/expats		1 444	1 444	1 444	1 444
Global cost of expats in Sudan	267 601				
Cost of expat/day	109				
**Total cost of expa for vacci**		**158 043**	**158 043**	**158 043**	**158 043**
Global cost of national staff	143 972				
**Cost of national staff for vacci**		**16 485**	**16 485**	**16 485**	**16 485**
**Cost of immunization materiel**		**554 665**	**2 183 041**	**649 600**	**457 920**
Global cost of vehicles in Sudan	101 907				
**Cost of vehicles for vacci**		**67 939**	**67 939**	**67 939**	**67 939**
**Cost of logistics for vacci**		**3 037**	**3 037**	**3 037**	**3 037**
**Cost of freight for vacci**		**96 959**	**96 959**	**96 959**	**96 959**
Global administrative cost	39 706				
**Administrative cost for vacci**		**19 853**	**19 853**	**19 853**	**19 853**
**TOTAL COSTS FOR VACCINATION**		**937 550**	**2 565 926**	**1 032 485**	**840 805**
**COST BY PERSON VACCINATED**		**0,49**	**1,34**	**0,54**	**0,44**

Nb of persons vaccinated =	persons vaccinated with MSF and MOH teams with vaccines mainly coming from MSF source
Nb of days/team =	number of days of immunization x number of teams on site
Cost of standard team/day =	standard team is 7 people. Per diem is 10000 SP x1(supervisor) and 5000 SP x6 (2 preparing,2 injecting, 2 registering) = **40 000** SP /team
Total cost of vacci. teams =	nb of days/team x cost of standard team
Nb of days/expats =	nb of day/expat allocated to immunization activities on a total of 2330 days/expat (see expat sheet) sharing urban & rural time is calculated from nb of days of vaccination (274 in urban) x mean of number of expats (2). Mean covers days of preparation. Taking 2 gives a total 40% time allocated to urban vacci & 60% time allocated to rural, according to interviews of expats.
Cost of expat/day =	global cost of mission divided by the total expat/day number (2330; see expat sheet)
Total cost of expa for vacci =	cost of one expat/day x nb of days/expat allocated to immunization activities
Cost of national staff for vacci =	global cost of national staff x 75%
Cost of immunization materiel =	global cost of vaccines + materiel less costs of ND & stocks
Cost of vehicles for vacci =	global cost of vehicles x 67%
Cost of freight for vacci =	given by packing list
Administrative cost for vacci =	global administrative cost x 50%

Results 2. Estimated costs of immunisation: bivalent vaccine vs. tetravalent vaccines ("full" and reduced dose)

ESTIMATED COST OF IMMUNIZATION

	SUDAN	Vaccine A+C	Vaccine A,C,Y,W	Vaccine A,C,Y,W	Vaccine A,C,Y,W
			Full dose	Reduced dose 1/5	Reduced dose 1/10
**Cost vaccine/dose in EURO**		**0,152**	**3,00**	**0,60**	**0,30**
**Nb of person vaccinated**		**1 916 802**	**1 916 802**	**1 916 802**	**1 916 802**
**Cost of immunization materiel**		**554 665**	**6 016 645**	**1 416 321**	**841 280**
**TOTAL COSTS FOR VACCINATION**		**937 550**	**6 399 530**	**1 799 206**	**1 224 165**
**COST BY PERSON VACCINATED**		**0,49**	**3,34**	**0,94**	**0,64**

These estimates of the cost of using fractional dose of trivalent vaccine (results 1) at 1/5 or 1/10 show clear reductions of cost by person vaccinated: respectively, 40% and 32% than the expected cost with the new trivalent vaccine. The cost per person vaccinated for 1/5 or 1/10 of the dose is at the same level as the bivalent A+C polysaccharide vaccine.

The benefit of reduced dose will be even higher, 28% and 19% than the expected cost with the tetravalent vaccine (results 2).

The calculations from the MSF intervention in an emergency context may have underestimated the real benefit of using a fractional dose.

Appendix 2 : Declaration of Helsinki

WORLD MEDICAL ASSOCIATION DECLARATION OF HELSINKI

Recommendations guiding physicians in
Biomedical research involving human subjects

Adopted by the 18th World Medical Assembly, Helsinki, Finland, June 1964,
Amended by the 29th World Medical Assembly, Tokyo, Japan, October 1975,
35th World Medical Assembly, Venice, Italy, October 1983
41st World Medical Assembly Hong Kong, September 1989
and the
48th General Assembly, Somerset West, Republic of South Africa, October 1996

INTRODUCTION

It is the mission of the physician to safeguard the health of the people. His or her knowledge and conscience are dedicated to the fulfilment of this mission.

The Declaration of Geneva of the World Medical Association binds the physician with the words, "The health of my patient will be my first consideration", and the International Code of Medical Ethics declares that, "A physician shall act only in the patient's interest when providing medical care which might have the effect of weakening the physical and mental condition of the patient."

The purpose of biomedical research involving human subjects must be to improve diagnostic, therapeutic and prophylactic procedures and the understanding of the aetiology and pathogenesis of disease.

In current medical practice most diagnostic, therapeutic or prophylactic procedures involve hazards. This applies especially to biomedical research.

Medical progress is based on research which ultimately must rest in part on experimentation involving human subjects.

In the field of biomedical research a fundamental distinction must be recognized between medical research in which the aim is essentially diagnostic or therapeutic for a patient, and medical research, the essential object of which is purely scientific and without implying direct diagnostic or therapeutic value to the person subjected to the research.

Special caution must be exercised in the conduct of research which may affect the environment, and the welfare of animals used for research must be respected.

Because it is essential that the results of laboratory experiments be applied to human beings to further scientific knowledge and to help suffering humanity, the World Medical Association has prepared the following recommendations as a guide to every physician in biomedical research involving human subjects. They should be kept under review in the future. It must be stressed that the standards as drafted are only a guide to physicians all over the world. Physicians are not relieved from criminal, civil and ethical responsibilities under the laws of their own countries.

I. BASIC PRINCIPLES

1. Biomedical research involving human subjects must conform to generally accepted scientific principles and should be based on adequately performed laboratory and animal experimentation and on a thorough knowledge of the scientific literature.

2. The design and performance of each experimental procedure involving human subjects should be clearly formulated in an experimental protocol which should be transmitted for consideration, comment and guidance to a specially appointed committee independent of the investigator and the sponsor provided that this independent committee is in conformity with the laws and regulations of the country in which the research experiment is performed.

3. Biomedical research involving human subjects should be conducted only by scientifically qualified persons and under the supervision of a clinically competent medical person. The responsibility for the human subject must always rest with a medically qualified person and never rest on the subject of the research, even though the subject has given his or her consent.

4. Biomedical research involving human subjects cannot legitimately be carried out unless the importance of the objective is in proportion to the inherent risk to the subject.

5. Every biomedical research project involving human subjects should be preceded with careful assessment of predictable risks in comparison with foreseeable benefits to the subject or to others. Concern for the interests of the subject must always prevail over the interests of science and society.

6. The right of the research subject to safeguard his or her integrity must always be respected. Every precaution should be taken to respect the privacy of the subject and to minimize the impact of the study on the subject's physical and mental integrity and on the personality of the subject.

7. Physicians should abstain from engaging in research projects involving human subjects unless they are satisfied that the hazards involved are believed to be predictable. Physicians should cease any investigation if the hazards are found to outweigh the potential benefits.

8. In publication of the results of his or her research, the physician is obliged to preserve the accuracy of the results. Reports of experimentation not in accordance with the principles laid down in this Declaration should not be accepted for publication.

9. In any research on human beings, each potential subject must be adequately informed of the aims, methods, anticipated benefits and potential hazards of the study and the discomfort it may entail. He or she should be informed that he or she is at liberty to abstain from participation in the study and that he or she is free to withdraw his or her consent to participation at any time. The physician should then obtain the subject's freely-given informed consent, preferably in writing.

10. When obtaining informed consent for the research project, the physician should be particularly cautious if the subject is in a dependent relationship to him or her or may consent under duress. In that case the informed consent should be obtained by a physician who is not engaged in the investigation and who is completely independent of this official relationship.

11. In case of legal incompetence, informed consent should be obtained from the legal guardian in accordance with national legislation. Where physical or mental incapacity makes it impossible to obtain informed consent, or when the subject is a minor, permission from the responsible relative replaces that of the subject in accordance with national legislation. Whenever the minor child is in fact able to give a consent, the minor's consent must be obtained in addition to the consent of the minor's legal guardian.

12. The research protocol should always contain a statement of the ethical considerations involved and should indicate that the principles enunciated in the present Declaration are complied with.

II. MEDICAL RESEARCH COMBINED WITH PROFESSIONAL CARE (Clinical Research)

1. In the treatment of the sick person, the physician must be free to use a new diagnostic and therapeutic measure, if in his or her judgement it offers hope of saving life, re-establishing health or alleviating suffering.

2. The potential benefits, hazards and discomfort of a new method should be weighed against the advantages of the best current diagnostic and therapeutic methods.

3. In any medical study, every patient - including those of a control group, if any - should be assured of the best proven diagnostic and therapeutic method. This does not exclude the use of inert placebo in studies where no proven diagnostic or therapeutic method exists.

4. The refusal of the patient to participate in a study must never interfere with the physician-patient relationship.

5. If the physician considers it essential not to obtain informed consent, the specific reasons for this proposal should be stated in the experimental protocol for transmission to the independent committee (I,2).

6. The physician can combine medical research with professional care, the objective being the acquisition of new medical knowledge, only to the extent that medical research is justified by its potential diagnostic or therapeutic value for the patient.

III. NON-THERAPEUTIC BIOMEDICAL RESEARCH INVOLVING HUMAN SUBJECTS (Non-clinical biomedical research)

1. In the purely scientific application of medical research carried out on a human being, it is the duty of the physician to remain the protector of the life and health of that person on whom biomedical research is being carried out.

2. The subjects should be volunteers -- either healthy persons or patients for whom the experimental design is not related to the patient's illness.

3. The investigator or the investigating team should discontinue the research if in his/her or their judgement it may, if continued, be harmful to the individual.

4. In research on man, the interest of science and society should never take precedence over considerations related to the well-being of the subject.

Appendix 3 : Menomune® – Aventis Pasteur technical form

AHFS Category 80:12

Caution: Federal (USA) law prohibits dispensing without prescription.

DESCRIPTION

Menomune® – A/C/Y/W-135, Meningococcal Polysaccharide Vaccine, Groups A, C, Y and W-135 Combined, for subcutaneous use, is a freeze-dried preparation of the group-specific polysaccharide antigens from *Neisseria meningitidis*, Group A, Group C, Group Y and Group W-135. *N. meningitidis* are cultivated with Mueller Hinton agar[1] and Watson Scherp[2] media. The purified polysaccharide is extracted from the *Neisseria meningitidis* cells and separated from the media by procedures which include centrifugation, detergent precipitation, alcohol precipitation, solvent or organic extraction and diafiltration. No preservative is added during manufacture.

The 0.78 mL vial of diluent contains sterile, preservative-free, pyrogen-free distilled water and is used for reconstitution of product supplied in 1 mL vials. The 6 mL vial of diluent contains sterile, pyrogen-free distilled water to which thimerosal (mercury derivative) 1:10,000 is added as a preservative. The 6 mL vial is for reconstitution of product supplied in 10 mL vials. After reconstitution with diluent as indicated on the label, the 0.5 mL dose is formulated to contain 50 µg of "isolated product" from each of Groups A, C, Y and W-135 in an isotonic sodium chloride solution.

Each dose of vaccine is also formulated to contain 2.5 mg to 5 mg of lactose added as a stabilizer [3]. The vaccine when reconstituted is a clear colorless liquid.

Potency is evaluated by measuring the molecular size of each polysaccharide component using a column chromatography method as standardized by the US Food and Drug Administration (FDA) and the World Health Organization (WHO)[4] for Meningococcal Polysaccharide Vaccine.

THIS VACCINE CONFORMS TO THE WORLD HEALTH ORGANIZATION (WHO) REQUIREMENTS

CLINICAL PHARMACOLOGY

N. meningitidis causes both endemic and epidemic disease, principally meningitis and meningococcemia. As a result of the control of *Haemophilus influenzae* type b infections, *N. meningitidis* has become the leading cause of bacterial meningitis in children and young adults in the United States (US), with an estimated 2,600 cases each year.[5,6] The case-fatality rate is 13% for meningitis disease (defined as the isolation of *N. meningitidis* from cerebrospinal fluid) and 11.5% for persons who have *N. meningitidis* isolated from blood,[5,6] despite therapy with antimicrobial agents (e.g., penicillin) to which US strains remain clinically sensitive.[5]

The incidence of meningococcal disease peaks in late winter to early spring. Based on multistate surveillance conducted during 1989 to 1991, serogroup B organisms accounted for 46% of all cases and serogroup C for 45%; serogroups W-135 and Y and strains that could not be serotyped accounted for most of the remaining cases.[5,6] Recent data indicate that the proportion of cases caused by serogroup Y strains is increasing.[5] In 1995, among the 30 states reporting supplemental data on culture-confirmed cases of meningococcal disease, serogroup Y accounted for 21% of cases.[7] Because of the success of *H. influenzae* type b vaccinations, the median age of persons with bacterial meningitis increased from 15 months in 1986 to 25 years in 1995.[8] The predominate organism causing meningitis in children 2 to 18 years of age is *N. meningitidis* based on 1995 surveillance data.[8] Serogroup A, which rarely causes disease in the US, is the most common cause of epidemics in Africa and Asia. A statewide serogroup B epidemic has been reported in the US.[9] Within the US, a vaccine for serogroup B is not yet available.

Outbreaks of serogroup C meningococcal disease (SCMD) have been occurring more frequently in the US since the early 1990s, and the use of vaccine to control these outbreaks has increased.[5] During 1980-1993, 21 outbreaks of SCMD were identified; eight of these occurred during 1992-1993.[10] Each of these 21 outbreaks involved from three to 45 cases of SCMD, and most outbreaks had attack rates exceeding 10 cases per 100,000 population, which is approximately 20 times higher than rates of endemic SCMD.[5] During 1981-1988, only 7,600 doses of

meningococcal vaccine were used to control four outbreaks; whereas, from January 1992 through June 1993, 180,000 doses of vaccine were used in response to eight outbreaks.[5]

Several discoveries impacted the future of meningococcal polysaccharide vaccines and demonstrated the significance of anti-capsular antibodies in protection.[11] In the late 1930s, serogroup-specific antigens of meningococcal serogroups A and C were identified as polysaccharides.[9] During the mid 1940s, investigators demonstrated that the protection of mice by anti-serogroup A meningococcal horse serum was directly related to its content of anti-polysaccharide antibodies.[11] Meningococcal polysaccharide vaccines were first demonstrated to be immunogenic in humans by Gotschlich and his co-workers in the 1960s when immunization of US Army recruits with serogroup A and C polysaccharides induced protective antibodies.[11] The investigators recorded a significantly reduced acquisition rate of serogroup C carriage among vaccinated recruits compared with unvaccinated individuals.[11]

Persons who have certain medical conditions are at increased risk for developing meningococcal infection. Meningococcal disease is particularly common among persons who have component deficiencies in the terminal common complement pathway (C3, C5-C9); many of these persons experience multiple episodes of infection.[5] Asplenic persons also may be at increased risk for acquiring meningococcal disease with particularly severe infections.[5] Persons who have other diseases associated with immunosuppression (e.g., human immunodeficiency virus [HIV] and *Streptococcus pneumoniae*) may be at higher risk for developing meningococcal disease and for disease caused by some other encapsulated bacteria.[5] Evidence suggests that HIV-infected persons are not at substantially increased risk for epidemic serogroup A meningococcal disease;[5] however, such patients may be at increased risk for sporadic meningococcal disease or disease caused by other meningococcal serogroups.[5] Previously, military recruits had high rates of meningococcal disease, particularly serogroup C disease; however, since the initiation of routine vaccination of recruits with bivalent A/C meningococcal vaccine in 1971, the high rates of meningococcal disease caused by those serogroups have decreased substantially and cases occur infrequently. [5]

A retrospective, epidemiological study was conducted in Maryland to compare the incidence of invasive meningococcal infection in college students with that of the general population of the same age. For the years 1992 to 1997, the incidence of meningococcal infection in Maryland college students was similar to the incidence of the general Maryland population of the same age. However, college students residing on-campus appeared to be at higher risk than those residing off campus.[12]

Vaccine efficacy. The immunogenicity and clinical efficacy of serogroups A and C meningococcal vaccines have been well established.[5] The serogroup A polysaccharide induces antibody in some children as young as 3 months of age, although a response comparable with that among adults is not achieved until 4 or 5 years of age; the serogroup C component is poorly immunogenic in recipients who are less than 18 to 24 months of age.[5] The serogroups A and C vaccines have demonstrated estimated clinical efficacies of 85% to 100% in older children and adults and are useful in controlling epidemics.[5] Serogroups Y and W-135 polysaccharides are safe and immunogenic in adults and in children greater than 2 years of age.[5] Although clinical protection has not been documented, vaccination with these polysaccharides induces bactericidal antibody. The antibody responses to each of the four polysaccharides in the quadrivalent vaccine are serogroup-specific and independent.[5]

Efficacy of serogroup A meningococcal vaccines was demonstrated in the 1970s in Africa and Finland, Egyptian school children aged 6 to 15 years showed 90% or greater protection during the first year after immunization with two different molecular sizes of serogroup A polysaccharide.[11] The higher molecular weight vaccine provided protection for at least three years.[11] In Finland, a randomized controlled mass immunization trial with serogroup A vaccine was conducted in response to a serogroup A epidemic. Results indicated 90 to 100% protection for three years.[11] In Rwanda, vaccination with bivalent A/C polysaccharide vaccine was performed in response to a serogroup A epidemic. A complete cessation of meningococcal disease was observed within two weeks of vaccination, yet the serogroup A carrier rate remained unchanged.[11]

Efficacy of serogroup C meningococcal vaccines was demonstrated in a field trial involving 20,000 troops in the US Army. Results suggested 90% efficacy under epidemic conditions which existed in basic training centers.[13] In Brazil, young children were vaccinated with serogroup C polysaccharide in response to a serogroup C epidemic. Results indicated that the vaccine was not effective in children under 24 months of age and only 52% effective in children aged 24 to 36 months.[11] However, studies suggested that the vaccine used in this trial was less immunogenic than other batches of similar vaccine that were used in US children; also, it was shown that the molecular size of the vaccine was smaller than the serogroup C polysaccharide in the present vaccine.[13] Thus, it is quite probable that the current serogroup C polysaccharide vaccine is more effective.[11]

A study performed using 4 lots of Menomune® – A/C/Y/W-135 in 150 adults showed at least a 4-fold increase in bactericidal antibodies to all groups in greater than 90 percent of the subjects.[14,15]

A study was conducted in 73 children 2 to 12 years of age. Post-immunization sera were not obtained on four children; seroconversion rates were calculated on 69 paired samples. Seroconversion rates as measured by bactericidal antibody were: Group A – 72%, Group C – 58%, Group Y – 90% and Group W-135 – 82%. Seroconversion rates as measured by a 2-fold rise in antibody titers based on Solid Phase Radioimmunoassay were: Group A – 99%, Group C – 99%, Group Y – 97% and Group W-135 – 89%.[16]

Duration of efficacy. Measurable levels of antibodies against the group A and C polysaccharides decrease markedly during the first 3 years following a single dose of vaccine.[5] This decrease in antibody occurs more rapidly in infants and young children than in adults. Similarly, although vaccine-induced clinical protection probably persists in schoolchildren and adults for at least 3 years, the efficacy of the group A vaccine in young children may decrease markedly with the passage of time. In a 3-year study, efficacy declined from greater than 90% to less than 10% among children who were less than 4 years of age at the time of vaccination, whereas among children who were greater than or equal to 4 years of age when vaccinated, efficacy was 67% 3 years later.[5,17] In a New Zealand study, children 2 to 13 years of age received a single dose of monovalent group A vaccine, 26% of children 3 to 23 months of age in this study received two doses of the vaccine, given approximately 3 months apart. After 2-1/2 years of active surveillance (1987 to 1989) there were no cases of invasive group A disease in children vaccinated at 2 years of age and older. [18]

INDICATIONS AND USAGE

Meningococcal Polysaccharide Vaccine, Groups A, C, Y and W-135 Combined, is indicated for active immunization against invasive meningococcal disease caused by these serogroups.[5]

Meningococcal Polysaccharide Vaccine, Groups A, C, Y and W-135 Combined may be used to prevent and control outbreaks of serogroup C meningococcal disease.[5]

For evaluation and management of suspected outbreaks, it is recommended that the health-care workers consult the MMWR for guidance.[5]

Routine vaccination is recommended for the following high-risk groups:[5]
1. Deficiencies in late Complement components (C3, C5-C9).
2. Functional or actual asplenia.
3. Persons with laboratory or industrial exposure to *N. meningitidis* aerosols.
4. Travelers to, and residents of, hyperendemic areas such as sub-Saharan Africa. For information concerning geographic areas for which vaccination is recommended, contact CDC at 404-332-4559.

The American College Health Association (ACHA) also recommends that college students consider vaccination to reduce the risk for potentially fatal meningococcal disease.[19]

Vaccinations also should be considered for household or institutional contacts of persons with meningococcal disease and for medical and laboratory personnel at risk of exposure to meningococcal disease.

This vaccine will not stimulate protection against infections caused by organisms other than Groups A, C, Y and W-135 meningococci.

Protective antibody levels may be achieved within 7 to 10 days after vaccination.[5]

Menomune® – A/C/Y/W-135 vaccine is not to be used for treatment of actual infection.

Menomune® – A/C/Y/W-135 vaccine will not protect against other etiologic agents, including *N. meningitidis* serogroup B, that cause meningitis.

Menomune® – A/C/Y/W-135 vaccine is not indicated for infants and children younger than 2 years of age except as short-term protection of infants 3 months and older against Group A.[11]

As with any vaccine, vaccination with Menomune ® – A/C/Y/W-135 *may not protect 100% of susceptible individuals.*

For persons remaining at high-risk, especially children who were first vaccinated at < 4 years of age, revaccination may be indicated.[5] (See **DOSAGE AND ADMINISTRATION** section.)

CONTRAINDICATIONS
Immunization should be deferred during the course of any acute illness.

IT IS A CONTRAINDICATION TO ADMINISTER MENOMUNE® – A/C/Y/W-135 TO INDIVIDUALS KNOWN TO BE SENSITIVE TO THIMEROSAL OR ANY OTHER COMPONENT OF THE VACCINE. FOR INDIVIDUALS SENSITIVE TO THIMEROSAL, ADMINISTER THE ONE DOSE PACKAGE SIZE AND RECONSTITUTE WITH THE 0.78 ML VIAL OF DILUENT THAT CONTAINS NO PRESERVATIVE.

WARNING This product contains dry natural latex rubber as follows: The stopper to the vial contains dry natural latex rubber.

If the vaccine is used in persons receiving immunosuppressive therapy, the expected immune response may not be obtained.

Menomune® – A/C/Y/W-135 should NOT be given at the same time as whole-cell pertussis or whole-cell typhoid vaccines due to combined endotoxin content. [0,221.]

PRECAUTIONS
GENERAL Care is to be taken by the health-care provider for the safe and effective use of Menomune® – A/C/Y/W-135.

EPINEPHRINE INJECTION (1:1000) MUST BE IMMEDIATELY AVAILABLE TO COMBAT UNEXPECTED ANAPHYLACTIC OR OTHER ALLERGIC REACTIONS.

Prior to an injection of any vaccine, all known precautions should be taken to prevent adverse reactions. This includes a review of the patient's history with respect to possible sensitivity to the vaccine or similar vaccines and to possible sensitivity to dry natural latex rubber.

Special care should be taken to avoid injecting the vaccine intradermally, intramuscularly, or intravenously since clinical studies have not been done to establish safety and efficacy of the vaccine using these routes of administration.

Health-care providers should obtain the previous immunization history of the vaccinee, and inquire about the current health status of the vaccinee.

A separate, sterile syringe and needle or a sterile disposable unit should be used for each patient to prevent transmission of hepatitis and other infectious agents from person to person. Needles should not be recapped and should be disposed of according to biohazard waste guidelines.

INFORMATION FOR PATIENT Patients, parents or guardians should be fully informed of the benefits and risks of immunization with Menomune ® – A/C/Y/W-135.

Patients, parents or guardians should be instructed to report any serious adverse reactions to their health-care provider.

As part of the patient's immunization record, the date, lot number and manufacturer of the vaccine administered should be recorded.[22,23,24]

DRUG INTERACTIONS If Menomune® – A/C/Y/W-135 is administered to immunosuppressed persons or persons receiving immunosuppressive therapy, an adequate immunologic response may not be obtained.

CARCINOGENESIS, MUTAGENESIS, IMPAIRMENT OF FERTILITY Menomune® – A/C/Y/W-135 has not been evaluated in animals for its carcinogenic, mutagenic potentials or impairment of fertility.

PREGNANCY REPRODUCTIVE STUDIES – PREGNANCY CATEGORY C Animal reproduction studies have not been conducted with Meningococcal Polysaccharide Vaccine, Groups A, C, Y and W-135. It is also not known whether Meningococcal Polysaccharide Vaccine, Groups A, C, Y and W-135 can cause fetal harm when administered to a pregnant woman or can affect reproduction capacity. Meningococcal Polysaccharide Vaccine, Groups A, C, Y and W-135 should be given to a pregnant woman only if clearly needed.
Although there is limited data, studies to date have found no evidence of teratogenicity of the polysaccharide quadrivalent meningococcal vaccine when given to pregnant women.[25]

NURSING MOTHERS It is not known whether this drug is excreted in human milk. Because many drugs are excreted in human milk, caution should be exercised when Menomune® – A/C/Y/W-135 is administered to a nursing woman.

PEDIATRIC USE

SAFETY AND EFFECTIVENESS OF MENOMUNE ® – A/C/Y/W-135 IN CHILDREN BELOW THE AGE OF 2 YEARS HAVE NOT BEEN ESTABLISHED.

ADVERSE REACTIONS

Adverse reactions to meningococcal vaccine are mild and consist principally of pain and redness at the injection site for 1 to 2 days. Pain at the site of injection is the most commonly reported adverse reaction, and a transient fever might develop in less than or equal to 2% of young children.[5]

Adverse events reported by 150 adults following vaccination with Menomune® – A/C/Y/W-135 are shown in Table 1.[14] The subjects were observed for three weeks following vaccination. Local reactions resolved within 48 hours and no significant systemic reactions were reported.[14]

ADVERSE EVENTS (%) FOLLOWING VACCINATION OF 150 ADULTS WITH MENOMUNE ® – A/C/Y/W-135 REACTIONS	MILD	MODERATE
**Local**		
Pain	2.6	2.0
Tenderness	36.0	9.0
Diameter	< 2 in.	≥2 in.
Erythema	3.8	1.2
Induration	4.4	1.2
**Systemic**		
Headaches	5.2	1.8
Malaise	2.5	0
Chills	2.5	0
Oral Temperature (°F)	2.6 (100–101)	0.6 (> 101)

In a clinical study involving 73 children 2 to 12 years of age, who received Menomune® – A/C/Y/W-135, local reactions consisting of erythema or tenderness were seen in approximately 40% of the children.[15] In another clinical study involving 53 children 4 to 6 years of age, who received Menomune® – A/C/Y/W-135, erythema was seen in 89% of the children, swelling in 92% and tenderness in 64%. None of these reactions were considered serious or necessitated medical intervention.[26]

On rare occasions, IgA nephropathy has occurred following vaccinations with Menomune® – A/C/Y/W-135. However, a cause and effect relationship has not been established.[16]

Menomune® – A/C/Y/W-135 should NOT be given at the same time as whole-cell pertussis or whole-cell typhoid vaccines due to combined endotoxin content.[20,21]

As with the administration of any vaccine, vaccine components can cause hypersensitivity reactions in some recipients.

Reporting of Adverse Events

The National Vaccine Injury Compensation Program, established by the National Childhood Vaccine Injury Act of 1986, requires physicians and other health-care providers who administer vaccines to maintain permanent vaccination records and to report occurrences of certain adverse events to the US Department of Health and Human Services. Reportable events include those listed in the Act for each vaccine and events specified in the package insert as contraindications to further doses of that vaccine.[22,23,24]

Reporting by patients, parents or guardians of all adverse events occurring after vaccine administration should be encouraged. Adverse events following immunization with vaccine should be reported by the health-care provider to the US Department of Health and Human Services (DHHS) Vaccine Adverse Event Reporting Systems (VAERS). Reporting forms and information about reporting requirements or completion of the form can be obtained from VAERS through a toll-free number 1-800-822-7967.[24]

Health-care providers also should report these events to the Pharmacovigilance Department, Aventis Pasteur Inc., Discovery Drive, Swiftwater, PA 18370 or call 1-800-822-2463.

DOSAGE AND ADMINISTRATION

Parenteral drug products should be inspected visually for extraneous particulate matter and/or discoloration prior to administration whenever solution and container permit. If either of these conditions exist, the vaccine should not be administered.

Reconstitute the vaccine using only the diluent supplied for this purpose. Draw the volume of diluent shown on the diluent label into a suitable size syringe and inject into the vial containing the vaccine. Shake vial until the vaccine is dissolved.

The immunizing dose is a single injection of 0.5 mL administered **subcutaneously**.

Special care should be taken to avoid injecting the vaccine intradermally, intramuscularly, or intravenously since clinical studies have not been done to establish safety and efficacy of the vaccine using these routes of administration.

Primary Immunization

For both adults and children, vaccine is administered subcutaneously as a single 0.5 mL dose. Protective antibody levels may be achieved within 7 to 10 days after vaccination.[5]

REVACCINATION

Revaccination of a single 0.5 mL dose administered subcutaneously may be indicated for individuals at high-risk of infection, particularly children who were first vaccinated when they were less than 4 years of age; such children should be considered for revaccination after 2 or 3 years if they remain at high-risk. Although the need for revaccination in older children and adults has not been determined, antibody levels decline rapidly over 2 to 3 years, and if indications still exist for immunization, revaccination may be considered within 3 to 5 years.[5,18]

Simultaneous administration of Menomune ® – A/C/Y/W-135 can be given concurrently with other vaccines at separate sites and separate syringes.[27] However, due to the combined endotoxin content, the vaccine should NOT be administered at the same time as whole-cell pertussis or whole-cell typhoid vaccines.[20,21] (See **WARNINGS** section.)

HOW SUPPLIED

Vial, 1 Dose, with 0.78 mL vial of diluent (contains NO preservative). Product No. 49281-489-01

Vial, 1 Dose (5 per package) with 0.78 mL vial of diluent (5 per package) (contains NO preservative). Product No. 49281-489-05

Vial, 10 Dose, with 6 mL vial of diluent (contains preservative) for administration with needle and syringe (NOT to be used with jet injector). Product No. 49281-489-91

STORAGE

Store freeze-dried vaccine and reconstituted vaccine, when not in use, between 2° – 8°C (35° – 46°F). Discard remainder of multidose vials of vaccine within 35 days after reconstitution. The single dose vial should be used within 30 minutes after reconstitution.

REFERENCES

1. Mueller, H, et al. A protein-free medium for primary isolation of the gonococcus and meningococcus. Proc Soc Exp Biol Med 48: 330, 1941
2. Watson, RG, et al. The specific hapten of group C (group IIa) meningococcus. II. Chemical nature. J Immunol. 81: 337, 1958
3. Tiesjema, RH, et al: Enhanced stability of meningococcal polysaccharide vaccines by using lactose as a menstruum for lyophilization. Bull WHO 55: 43-48, 1977
4. WHO Technical Report Series, No. 658, 1981
5. Recommendation of the Advisory Committee on Immunization Practices (ACIP). Control and prevention of meningococcal disease and control and prevention of serogroup C meningococcal disease: evaluation and management of suspected outbreaks. MMWR 46: No. RR-5, 1997
6. CDC. Laboratory-based surveillance for meningococcal disease in selected areas – United States, 1989-1991. MMWR 42: No SS-2, 1993
7. CDC. Serogroup Y Meningococcal Disease – Illinois, Connecticut, and Selected Areas, United States, 1989-1996. MMWR 46: Vol. 45, 1010-1013, 1996
8. Schuchat, A, et al. Bacterial Meningitis in the United States in 1995. N Eng J Med 337: 970-976, 1997
9. CDC. Serogroup B meningococcal disease – Oregon 1994. MMWR 44: 121-124, 1995
10. Jackson, LA, et al. Serogroup C meningococcal outbreaks in the United States: an emerging threat. JAMA 273: 383-389, 1995

11. Frasch, CE. Meningococcal vaccines; past, present and future, in Meningococcal Disease, ed. K. Cartwright. John Wiley and Sons Ltd, 1995
12. Harrison LH, et al. Risk of meningococcal infection in college students. JAMA 281: 1906-1910, 1999
13. Lepow, ML. Meningococcal vaccines, in Vaccines, ed. SA Plotkin and EA Mortimer. WB Saunders Co., 1994
14. Hankins, WA, et al: Clinical and serological evaluation of a Meningococcal Polysaccharide Vaccine Groups A, C, Y and W-135. Proc Soc Exper Biol Med 169: 54-57, 1982
15. Lepow, ML, et al: Reactogenicity and immunogenicity of a quadrivalent combined meningococcal polysaccharide vaccine in children. J Infect Dis 154: 1033-1036, 1986
16. Unpublished data available from Aventis Pasteur Inc.
17. Reingold, AL, et al: Age-specific differences in duration of clinical protection after vaccination with meningococcal polysaccharide A vaccine. Lancet. No. 8447: 114-118, 1985
18. Lennon, D, et al: Successful intervention in a Group A meningococcal outbreak in Auckland, New Zealand. Pediatr Infect Dis J 11: 617-623, 1992
19. Collins, MJ, et al. Student Health Centers urged to alert students to danger of the disease and provide campus vaccination programs. American College Health Association (ACHA), Baltimore, MD, Press Release 1997
20. Kuronen, T, et al. Adverse reactions and endotoxin content of polysaccharide vaccines. Develop Biol Standard, Vol. 34: 117-125, 1977
21. Peltola, H., et al. Meningococcus group A vaccine in children three months to five years of age: adverse reactions and immunogenicity related to endotoxin content and molecular weight of the polysaccharide. J Pediatr Vol 92: No 5, 818-822, 1978
22. CDC. National Childhood Vaccine Injury Act: requirements for permanent vaccination records and for reporting of selected events after vaccination. MMWR 37: 197-200, 1988
23. Food and Drug Administration. New reporting requirements for vaccine adverse events. FDA Drug Bull 18 (2), 16-18, 1988
24. CDC. Vaccine Adverse Event Reporting System – United States. MMWR 39: 730-733, 1990
25. Letson, GW, et al. Meningococcal vaccine in pregnancy: an assessment of infant risk. Pediatr Infect Dis J 17(3), 261-263, 1998
26. Scheifele, DW, et al. Local adverse effects of meningococcal vaccine. Can Med Assoc J 150: 14-15, 1994
27. American Academy of Pediatrics, Meningococcal infections. In: Peter G, ed. 1997 Red Book: Report of the Committee on Infectious Diseases. 24th ed. Elk Grove Village, IL: American Academy of Pediatrics; 361, 1997.

Manufactured by:
Aventis Pasteur Inc.
Swiftwater PA 18370

Product information
as of January 2003
USA Printed in USA

Aventis Pasteur

Aventis

4812/4813

Appendix 4 : Estimated Ugandan Population aged 2 to 20, from the 2002/03 Uganda National Household Survey.

Uganda Bureau of Statistics, February 2004.

Age	Number	% Total	Number of patients to be included in the study
2	970 133	6,7	48
3	990 583	6,8	49
4	1 082 578	7,5	54
5	859 565	5,9	43
6	1 010 011	7,0	50
7	844 760	5,8	42
8	961 627	6,6	48
9	777 933	5,4	39
10	934 363	6,5	46
11	588 067	4,1	29
12	935 060	6,5	47
13	709 573	4,9	35
14	708 895	4,9	35
15	536 283	3,7	27
16	573 305	4,0	29
17	408 541	2,8	20
18	579 822	4,0	29
19	372 486	2,6	19
20	625 030	4,3	31
**Total**	**14 468 615**	**100**	**720**

Appendix 5 : Informed consent statement

Study purpose : Investigators from Epicentre and Mbarara University would like you/your child to participate in a study to find out if three different doses of the meningitis vaccine give the same level of protection against meningitis in this community. The decision on the dose of vaccine you or your child receives will be based on a random list. We would also like to determine the proportion of people in this community who are carriers of the organisms that cause meningitis and those who have natural immunity against meningitis. 720 healthy volunteers will participate in this study and will be followed for a period 2 years.

Study process : Three groups will be made based on the vaccine dosage. You/your child will be randomly allocated to one of these groups without you/your child knowing to which group you/he/she are/is assigned. At the beginning of the study the investigator will ask you/your child questions about your/his/her general health and do a medical examination. We shall also take off a sample of mucus from your/your child's throat to determine whether you have or your child has organisms that cause meningitis and a sample of blood to measure your/your child's natural immunity to meningitis. You/your child will then be given the vaccine by injection. After 1 month, we shall take a second sample of mucus from your/your child's throat and a second blood sample to determine the response to the vaccine.

Based on a random decision you/your child may be given a second vaccination after 1 year. Should this be the case, you/your child will also be asked to give 2 more blood samples, one before the second vaccination and the other 1 month later to determine the your/your child's immune response to the second dose of the vaccine.

After 2 years, we shall take the final blood sample from you/your child to determine the level of immunity to meningitis after this period.

This vaccine will not be given to pregnant or breastfeeding women as the immune response during this period is modified. Therefore before being included in the study, we will ask you to have a pregnancy test done to make sure that you are not pregnant.

Confidentiality : Scientists from the Norwegian Institute of Public Health (the providers of technical assistance), the World Health Organisation, the ethics committees or the regulatory authorities may look at your records. Your medical records will be kept confidential. All records used for the results of the study will not have your name on them.

Risks and benefits from participating in the study : Neighbouring countries such as Burundi and Rwanda have faced in 2002 and 2003 outbreaks of meningitis which led to mass vaccination campaign in the affected areas. By participating in this study, you/your child will be protected against meningitis in case an outbreak occurred. In addition, you will help medical doctors to decide what is the best dose of vaccine that will offer protection and control future meningitis outbreaks.

The vaccine used for the study is well known for more than 20 years in the United States and in Europe. Adverse events can occur such as mild redness, swelling or tenderness at the injection site but they remain rare and last less than 48 hours. As with the administration of any vaccine, vaccine components can cause hypersensitivity reactions in some patients and precautions will be taken by the doctors for the safe use of the vaccine for you/your child.

Further more, you/your child will receive an mosquito net and we will refund your transport from your home to the vaccine site or to Mbarara hospital when necessary.

Withdrawal from the study and compensation : You can ask the doctor any questions about the study, the meningitis disease and the vaccine product. Your participation in this study is entirely voluntary and you are free to withdraw at any time. Your withdraw from this study will not prejudice to your care or your rights to receive a routine treatment at the health unit.
Your doctor may also withdraw you from the study if it is in your best interests (e.g. health condition not compatible with immunization). We will keep you informed if new information concerning this vaccine and your health arise during the study. If you become sick or are injured as a result of the participation in this study, Epicentre will provide medical treatment and pay the reasonable costs of such treatment. In such an event we are based in the Mbarara Hospital if you want to contact us. Please find Dr Laurence Ahoua, Dr Francis Bajunirwe, Ms Carole Fogg or ring telephone number 077 721 748/9.

I have read and understood the above information and my questions have been answered to my satisfaction. I give voluntary consent to my participation in this study.

I understand that I am free to withdraw from the study at any time and that I will continue to receive the care I am entitled to.

Patient's Name
Patient's signature/fingerprint Date

Investigator's Name
Investigator's Signature Date

If oral consent given:
Name and signature of witness: Date

Not to be quoted or distributed
Confidential

EKIHANDIKO KYOKUSHARAMU OYEKUNDIIRE

EKIGYENDERERWA KY'OKUCONDOOZA

Abacondoozi kuruga omu kigombe kya EPICENTRE hamwe na Yunivaasite ya Mbarara nibakushaba nari omwana waawe, kwetaba omu kucondooza okwine ekigyendererwa ky'okumanya yaaba Doozi ishatu zitarikushushana z'omubazi gw'okusirika endwara y'omuraramo, nizibaasa kutambira abantu abatwire omu kyanga eki.

Okusharamu ahakipimo kyomubazi ogu oratungye ninga omwana waawe nikuza kusharwamu akaruru. Ekindi kintu kikuru eki turikwenda kumanya, n'omuhendo gw'abantu abatwire omu kyanga ekya Mbarara abarikureebeka baine amagara marungi kwonka baine obukooko oburikurwaza omuraramo omu shagama yaabo, hamwe n'abo abaine abaserukare abarikukingira endwara y'omuraramo omu shagama yabo.

Abantu magana mushanju na makumi abiri [720] abaine amagara marungi kandi abeekundiire, nibo barikuza kwetaba omu kucondooza n'okugyezibwa kw'omubazi ogu. Abantu aba, nibaza kwecumintirizibwa kuheza emyaka ebiri.

OKUCONDOOZA OKU KURAATWAZIBWE

Abantu abaraagyezibwe omubazi gw'okusirika omuraramo, nibaza kuba bari omu bicweka bishatu. Iwe nari omwana waawe n'oza kuza omuri kimwe aha bicweka ebyo bishatu. Kwonka iwe nari omwana waawe tihaine orikuba namanya ekicweka ekyarimu.

Aha kutandika kucondooza enkora y'omubazi gw'okusirika omuraramo, omucondoozi naabanza yaakubuza ebibuzo ebikwataine namagara gawe ninga ago'omwana waawe, abakyebere kwenda kumanya yaaba mwine amagara marungi. Omu kukyebera, nitukwihaho ekikonda aha maraka nari aha g'omwana waawe ahabw'okwenda kukikyebera n'okumanya yaaba oine obukooko oburikurwaza omuraramo nari yaaba omwana waawe niwe abwine. Nituza n'okukwihaho eshagama nari ey'omwana waawe, kugikyebera ahabw'okwenda kumanya yaaba aine ninga oine abaserukare abarikukingira omuraramo.

Bwanyima y'okumanya ebyaruga omukukyebera, iwe nari omwana waawe naija kuterwa akakatu komubazi gw'okusirika omuraramo. Ku haraahweho okwezi kumwe, nitwiha ekikonda aha maraka gaawe nari ag'omwana waawe, hamwe n'eshagama omurundi gwa kabiri, ahabw'okwenda kumanya yaaba omubazi gw'okusirika omuraramo nigureta ninga nigwongyera ahabusirikare bwokutanga omuraramo.

Bwanyima y'omwaka gumwe, iwe nari omwana waawe, n'obasa kuheebwa dozi yakabiri y'omubazi gw'okusirika omuraramo kandi nabwo tihariho kumanya iwe ninga omwana wawe orahebwe omubazi. Ku orikuhebwa omubazi omurundi gwa kabiri, iwe nari omwana waawe noija kushabwa kwihwaho eshagama y'okukyebera emirundi ebiri; ogw'okubanza otakateirwe ekikatu ky'okusirika omuraramo kandi ogwa kabiri, noogutunga hahweireho okwezi kumwe, kwenda kumanya yaaba omubazi ogwa doozi ya kabiri nigureta ninga nigwongyera ahabusirikare bwokutanga omuraramo.

Aha muheru y'emyaka ebiri, nitukyebera omurundi gw'okuhereerukayo eshagama yaawe nari ey'omwana waawe, okwenda kumanya yaaba oine abaserukare abaine amaani g'okukingira endwara y'omuraramo.

ABAKAZI ABAINE ENDA

Omubazi ogu tigurahebwe abakazi abaine enda nari abarikwontsya ahakuba abaserukare abari omu shagama yaabo nibaba bataine maani. N'ahabw'ekyo buri mukazi nari omwishiki atakeetabire omu kucoondoza oku, n'okubanza twamushaba kwikiriza kukyeberwa yaaba ataine nda, reero akabona kwikirizibwa.

OBWESIGYE/ENAAMA

Abakugu omu bya Saayansi abarikukomooka Norway omu itendekyero rye byamagara gabantu [abarikuheereza abacondoozi ba Epicentre/Mbarara University obuhwezi], ab'ekitongore ky'eby'amagara eky'Amahanga Ageeteeraine [*World Health Organisation* - WHO] , ab'obukiiko oburikurinda emicwe n'emitwarize y'abacondoozi hamwe n'abandi beebembezi b'ebitongore ebiine obujunaanizibwa bw'okureeba ngu okucondooza kw'omubazi ogu kwagyendera aha mateeka, tihaine obu bariba baikiriize ngu hagire owaata aha mushana eiziina ryawe n'ebirugire omu kukyebera. Eiziina ryawe niriguma riri ekihama.

AKABI/AMAGOBA EBIKURUGA OMUKWETABA OMU KUCONDOOZA OKU

Endwara y'omuraramo ekabarukaho omu mahanga gamwe agaturi haihi nka Rwanda na Burundi omu mwaka 2002 na 2003; reero kyatuma haabaho ehururu y'okuteera abantu b'ebyanga ebyo ebikatu by'okukingira endwara egi. Ku orikuba oyetabire omu kucondooza oku, obundi endwara y'omuraramo yaabarukaho terikukukwata, ahakuba nooba waasirikirwe. Okwongyera ahari eki, ku orikuba oyetabire omu kucondooza oku, noohwera abashaho kumanya ekipimo [doozi] ky'omubazi ogurikukingira endwara y'omuraramo kandi kihwera n'okuzibira okujanjaara kw'omuraramo omu biro by'omumaisho.

Omubazi ogurikwejunisibwa omu kucondooza oku, gumazire emyaka erikurenga makumi abiri [20 years] nigumanywa kandi nigukozesibwa omuri'Amerika na Buraaya. Obundi bakuteera omubazi ogu, omubiri gwawe nigubaasa kuhindukaho, gutukure, hagire ahaazimba nari ahu baateera ekikatu hoorobe, kwonka n'ekitari kya butoosha kandi omu biro bibiri [eshaaha 48] nooba waabaire gye. Abashaho nibeegyendesereza munonga kureeba ngu omubazi ogu gwakozesibwa gye.

Okwongyerera aha kusirikwa kw'endwara y'omuraramo, iwe nari omwana waawe, nibabaheereza akatimba akarikutanga ensiri ezirikujanjaaza obukooko oburikurwaza omushwija. N'empiiha z'engyendo okuruga omuka kwija ahu barikuhera omubazi nari kwija omu irwariro rya Mbarara, n'okugaruka omuka, nizikugarurirwa hoona ahu kirikuba nikyetaagisa.

OKURUGA OMU'MUSHOMO NA NO'KUSHUMBUSHUBWA

Eky'okwetaba omu kucondooza kw'enkora y'omubazi gw'okusirika omuraramo kiri aha kukunda kwawe. Tihaine muntu n'omwe orikukugyema kandi na waayenda kukireka, oine obugabe kukirugamu okataaha obwire bwona obu oraayendere. Otakeetabire omu by'okucondooza oku, oine obugabe kubuuza omushaho byona ebi waakubaasa kuba nooyenda kumanya ebikwatiraine n'okucondooza endwara y'omuraramo hamwe n'omubazi gw'okugisirika.

Okugira ngu waareka okwetaba omu by'okucondooza, tikirikumanyisa ngu ab'eirwariro nibaija kwanga kukuragurira nk'oku obutoosha barikutwariza abandi. Nangwa n'omushaho waawe naabaasa kukwiha omu by'okwetaba omu kucondooza oku, yaareeba ngu amagara gaawe nigabaasa kushiishwa omubazi ogurikuba nigugyezibwa.

Ku harikugira ekintu kisya ekyabaho kikwatiraine n'omubazi ogu, omu bwire bw'okucondooza nari ekiine akakwate n'amagara gaawe, nitukumanyisa.

Ku waakurwara nari okahutaara omu bunaku bw'okugyezibwa omubazi ogu, EPICENTRE nekujanjaba hataine sente ezi oshashwire kandi na haagira sente ezi waashohoza aha kujanjabwa kwawe, nibazikugaruriza, zaaba zitari z'omuhendo ogurengaine.

Kuri noorwara nari okahutaara, nootuhika omu irwariro rya Mbarara. Noohikira nari nooreebana n'abaayorekwa ahaifo: DR. LAURENCE AHOUA, DR. FRANCIS BAJUNIRWE, OMUKY. CAROLE FOGG nari oteere esimu 077-721748/9.

Naashoma kandi naayetegyereza. N'ebi naabuuza baabingarukiramu gye, naananuka. Naayehayo kandi nikiriza kwetaba omu by'okucondooza omubazi gw'okusirika omuraramo.

Ninkimanya ngu obwire bwona nyine obugabe bw'okuruga omu by'okucondooza okuriho kandi tikirikunyihaho obugabe bwangye bw'okutunga obujanjabi.

- **EZIINA RY'OMURWAIRE: ………………………………EBIRO ………………..**

OMUKONO GW'OMURWAIRE NARI EKINKUMU ………………………………

- **EIZIINA RY'OMUCONDOOZI………………………………EBIRO …………….**

OMUKONO GW'OMUCONDOOZI ………………………………………………….

- **EZIINA RY'OMWEMA AHABW'ORIKWIKIRIZA ATEEHANDIKIIRE ………………………………………………….EBIRO ………………………………..**

OMUKONO GW'OMWEMA ……………………………………………………………

Appendix 6 : Case Report Form (CRF)

STUDY SITE	Country : *Uganda*	District : *Mbarara*	County :	Subcounty :	Parish :	Town/Village :
	Principal investigators :		Health facility's name :		Immunization site :	
PATIENT	**Inclusion number :**	Age (months or years) :	Sex (M/F) :	Weight (kg) :	Height (cm) :	

ELIGIBILITY CRITERIA : Does the patient meet entry criteria ? Answer questions below

Inclusion [ALL MUST be YES]

- Age between 2 and 20 years old Y N
- Resident in Mbarara district and with 15 km Y N
- No planning of moving from area in the next 2 years Y N
- Available for follow-up for 2 years Y N
- Normal respiratory system examination Y N
- Normal medical examination Y N
- Informed consent signed Y N

Exclusion [ALL MUST be NO]

- Allergy (dry natural latex or other) Y N
- Whole cell-pertussis vaccine ≤ 1 month Y N
- Whole-cell typhoid vaccine ≤ 1 month Y N
- Pregnancy or Breastfeeding Y N
- Severe chronic disease (e.g. TB…)
- Known congenital / acquired immunodeficiency (HIV) Y N
- Current acute illness or infection of the skin (rashes), ear, nose or throat Y N
- Malnutrition signs Y N

REGISTRATION : if all Inclusion criteria are answered YES and all Exclusion criteria NO, then

1. Attach informed consent
2. Open randomisation envelope
3. Allocate to Vaccine group according to randomisation list and assign the patient Inclusion number

Person carrying out inclusion process :

Signature :

Date :

VACCINE GROUP ALLOCATION TO :

Group		Sub-group	
n° 1 (dose 50 µg)	☐	A	☐
n° 2 (dose 10 µg)	☐	B	☐
n° 3 (dose 5 µg)	☐		

Not to be quoted or distributed

Confidential

PATIENT	Inclusion number :	Group n°______		Sub-group _____	
Time table	Day 0 (0h)<br>(date : ___/___/___)	Week 4<br>(date : ___/___/___)	Month 12<br>(date : ___/___/___)	Month 13<br>(date : ___/___/___)	Year 2<br>(date : ___/___/___)
Was the patient seen (Y/N)<br>If NO, precise the reason :					
CLINICAL EXAMINATION :<br>- Axillary T° (C°)<br>- Arterial tension<br>- Rash (Y/N)<br>- Others :					
CLINICIAN ID :					

NOTES :

....

....

....

....

....

....

Time table	Day 0<br>(date : ___/___/___)	Week 4<br>(date : ___/___/___)	Month 12<br>(date : ___/___/___)	Month 13<br>(date : ___/___/___)	Year 2<br>(date : ___/___/___)
**ADVERSE EVENTS (Y/N)**<br>If Y, specify type **AE n°1**:<br>Date start :<br>Date end :<br>Severity :<br>Vaccine-event relationship :<br>Outcome :<br>Treatment :	 1)__________________<br>____/____/____<br>____/____/____<br>__________<br>__________<br>__________<br>__________	 ____________________<br>____/____/____<br>____/____/____<br>__________<br>__________<br>__________<br>__________	 ____________________<br>____/____/____<br>____/____/____<br>__________<br>__________<br>__________<br>__________	 ____________________<br>____/____/____<br>____/____/____<br>__________<br>__________<br>__________<br>__________	 ____________________<br>____/____/____<br>____/____/____<br>__________<br>__________<br>__________<br>__________
If Y, specify type **AE n°2** :<br>Date start :<br>Date end :<br>Severity :<br>Vaccine-event relationship :<br>Outcome :<br>Treatment :	2)__________________<br>____/____/____<br>____/____/____<br>__________<br>__________<br>__________<br>__________	____________________<br>____/____/____<br>____/____/____<br>__________<br>__________<br>__________<br>__________	____________________<br>____/____/____<br>____/____/____<br>__________<br>__________<br>__________<br>__________	____________________<br>____/____/____<br>____/____/____<br>__________<br>__________<br>__________<br>__________	____________________<br>____/____/____<br>____/____/____<br>__________<br>__________<br>__________<br>__________
If Y, specify type **AE n°3** :<br>Date start :<br>Date end :<br>Severity :<br>Vaccine-event relationship :<br>Outcome :<br>Treatment :	3)__________________<br>____/____/____<br>____/____/____<br>__________<br>__________<br>__________<br>__________	___________________<br>____/____/____<br>____/____/____<br>__________<br>__________<br>__________<br>__________	____________________<br>____/____/____<br>____/____/____<br>__________<br>__________<br>__________<br>__________	____________________<br>____/____/____<br>____/____/____<br>__________<br>__________<br>__________<br>__________	____________________<br>____/____/____<br>____/____/____<br>__________<br>__________<br>__________<br>__________
Observation for AEs :					

Severity : 1=mild ; 2= moderate ; 3= severe ; 4 =very severe
Vaccine-event relationship : 1=not related ; 2=unlikely ; 3 = possible; 4 = probable; 5= definite ; 6= Unknown
Outcome : 1=recovery ; 2= still present ; 3= sequelae ; 4= death ; 5= unknown
Treatment : 1= No treatment ; 2= Out patient treatment ; 3= Hospitalised

Time table	Day 0<br>(date : ___/___/___)	Week 4<br>(date : ___/___/___)	Month 12<br>(date : ___/___/___)	Month 13<br>(date : ___/___/___)	Year 2<br>(date : ___/___/___)
**SERIOUS ADVERSE EVENTS (Y/N)** (If YES, inform PI)					
If Y, specify type **SAE n°1**:<br>Date start :<br>Date end :<br>Severity :<br>Vaccine-event relationship :<br>Outcome :<br>Treatment :	1)__________________<br>____/____/____<br>____/____/____<br>__________<br>__________<br>__________<br>__________	__________________<br>____/____/____<br>____/____/____<br>__________<br>__________<br>__________<br>__________	__________________<br>____/____/____<br>____/____/____<br>__________<br>__________<br>__________<br>__________	__________________<br>____/____/____<br>____/____/____<br>__________<br>__________<br>__________<br>__________	__________________<br>____/____/____<br>____/____/____<br>__________<br>__________<br>__________<br>__________
If Y, specify type **SAE n°2** :<br>Date start :<br>Date end :<br>Severity :<br>Vaccine-event relationship :<br>Outcome :<br>Treatment :	2)__________________<br>____/____/____<br>____/____/____<br>__________<br>__________<br>__________<br>__________	__________________<br>____/____/____<br>____/____/____<br>__________<br>__________<br>__________<br>__________	__________________<br>____/____/____<br>____/____/____<br>__________<br>__________<br>__________<br>__________	__________________<br>____/____/____<br>____/____/____<br>__________<br>__________<br>__________<br>__________	__________________<br>____/____/____<br>____/____/____<br>__________<br>__________<br>__________<br>__________
If Y, specify type **SAE n°3** :<br>Date start :<br>Date end :<br>Severity :<br>Vaccine-event relationship :<br>Outcome :<br>Treatment :	3)__________________<br>____/____/____<br>____/____/____<br>__________<br>__________<br>__________<br>__________	__________________<br>____/____/____<br>____/____/____<br>__________<br>__________<br>__________<br>__________	__________________<br>____/____/____<br>____/____/____<br>__________<br>__________<br>__________<br>__________	__________________<br>____/____/____<br>____/____/____<br>__________<br>__________<br>__________<br>__________	__________________<br>____/____/____<br>____/____/____<br>__________<br>__________<br>__________<br>__________
Observation for SAEs :					

Severity : 1=mild ; 2= moderate ; 3= severe ; 4 =very severe
Vaccine-event relationship : 1=not related ; 2=unlikely ; 3 = possible; 4 = probable; 5= definite ; 6= Unknown
Outcome : 1=recovery ; 2= still present ; 3= sequelae ; 4= death ; 5= unknown
Treatment : 1= No treatment ; 2= Out patient treatment ; 3= Hospitalised

PATIENT	Inclusion number :	Group n°______		Sub-group _____	
MENINGOCOCCAL VACCINE DRUG	Drug name :	Manufacturer :	Lot number :	Expiry date :	Vaccine dose (µg) :
Time table	Day 0<br>(date : ___/___/___)	Week 4<br>(date : ___/___/___)	Month 12<br>(date : ___/___/___)	Month 13<br>(date : ___/___/___)	Year 2<br>(date : ___/___/___)
Vaccine dose injected (µg) :		X		X	X
Serology :<br>- ELISA IgG (A)<br>- ELISA IgG (C)<br>- ELISA IgG (Y)<br>- ELISA IgG (W135)	 -<br>-<br>-<br>-	 -<br>-<br>-<br>-	*(only subgroup B)*<br>-<br>-<br>-<br>-	*(only subgroup B)*<br>-<br>-<br>-<br>-	 -<br>-<br>-<br>-
- SBA IgG (A)<br>- SBA IgG (C)<br>- SBA IgG (Y)<br>- SBA IgG (W135)	-<br>-<br>-<br>-	-<br>-<br>-<br>-	-<br>-<br>-<br>-	-<br>-<br>-<br>-	-<br>-<br>-<br>-
Pharyngeal carriage :			X	X	X

Overall assessment : Adequate serological response (ASR) *N. meningitidis* pharyngeal carrier (CARR) Withdrawn (WTH) Lost to follow-up (LOSS)

Reason for WTH :

Day of assessment :

Patient confidential information : keep separately from the Case Report Form

<table>
<tr><td>STUDY SITE</td><td>Health facility's name :</td><td>District :</td><td>County :</td><td>Subcounty :</td><td>Parish :</td><td>Town/Village :</td></tr>
<tr><td rowspan="3">PATIENT</td><td colspan="2">Inclusion number :</td><td>Health Card number :</td><td colspan="3">First name :

Last name :</td></tr>
<tr><td>Age (months or years) :</td><td>Sex (M/F) :</td><td colspan="4">Contact home address (be as precise as possible) :</td></tr>
<tr><td colspan="2">Local council Chairman (LC 1) :</td><td colspan="2">Name of guardian :</td><td colspan="2">Name of CHW :</td></tr>
<tr><td colspan="2">GROUP ALLOCATION :</td><td colspan="2">Group (1/ 2/ 3) :</td><td colspan="3">Subgroup (A / B) :</td></tr>
</table>
